# Continuous flow synthesis of pyridinium salts accelerated by multi-objective Bayesian optimization with active learning†

**DOI:** 10.1039/d3sc01303k

**Published:** 2023-07-12

**Authors:** John H. Dunlap, Jeffrey G. Ethier, Amelia A. Putnam-Neeb, Sanjay Iyer, Shao-Xiong Lennon Luo, Haosheng Feng, Jose Antonio Garrido Torres, Abigail G. Doyle, Timothy M. Swager, Richard A. Vaia, Peter Mirau, Christopher A. Crouse, Luke A. Baldwin

**Affiliations:** a Materials and Manufacturing Directorate, Air Force Research Laboratory Wright-Patterson AFB OH 45433 USA luke.baldwin.1@us.af.mil; b UES, Inc. Dayton OH 45431 USA; c National Research Council Research Associate, Air Force Research Laboratory Wright-Patterson AFB OH 45433 USA; d Department of Chemistry, Purdue University West Lafayette IN 47907 USA; e Department of Chemistry, Massachusetts Institute of Technology Cambridge MA 02139 USA; f Department of Chemistry, Princeton University Princeton NJ 08544 USA; g Department of Chemistry and Biochemistry, University of California Los Angeles CA 90095 USA

## Abstract

We report a human-in-the-loop implementation of the multi-objective experimental design *via* a Bayesian optimization platform (EDBO+) towards the optimization of butylpyridinium bromide synthesis under continuous flow conditions. The algorithm simultaneously optimized reaction yield and production rate (or space-time yield) and generated a well defined Pareto front. The versatility of EDBO+ was demonstrated by expanding the reaction space mid-campaign by increasing the upper temperature limit. Incorporation of continuous flow techniques enabled improved control over reaction parameters compared to common batch chemistry processes, while providing a route towards future automated syntheses and improved scalability. To that end, we applied the open-source Python module, nmrglue, for semi-automated nuclear magnetic resonance (NMR) spectroscopy analysis, and compared the acquired outputs against those obtained through manual processing methods from spectra collected on both low-field (60 MHz) and high-field (400 MHz) NMR spectrometers. The EDBO+ based model was retrained with these four different datasets and the resulting Pareto front predictions provided insight into the effect of data analysis on model predictions. Finally, quaternization of poly(4-vinylpyridine) with bromobutane illustrated the extension of continuous flow chemistry to synthesize functional materials.

## Abstract 2

Human-in-the-loop experimentation enables interactive machine learning for continuous flow chemistry reaction planning and optimization.

## Graphical abstract

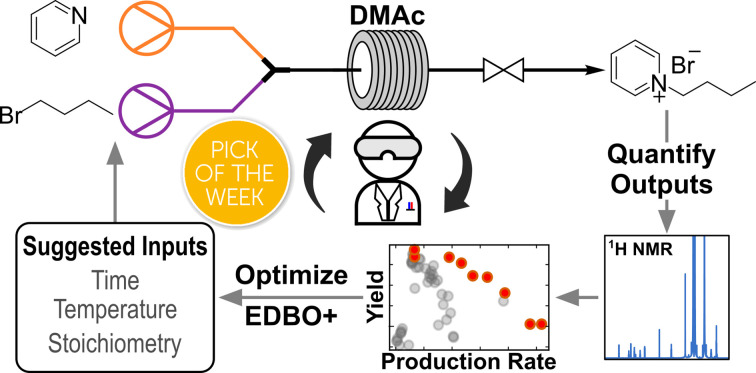

## Introduction

The optimization of chemical reactions has long relied upon a chemist's intuition and ability to evaluate multiple parameters within a predefined reaction space. In an optimization campaign, solvent, concentration, stoichiometry, temperature, and time must be considered, but the effects of each variable are typically evaluated individually and systematically. To evaluate the impact of these variables, single-objective optimization models have been developed that target a global optimal solution.^1–3^ Although effective for reaction campaigns targeting one objective (*e.g.*, maximizing yield), the primary limitation of single-objective optimizers is the inability to solve multiple reaction goals simultaneously. Recent advances in multi-objective optimizers have facilitated the optimization of complex multidimensional problems.^3–6^ To determine the ideal conditions for a chemical reaction, or synthesis, it is advantageous to incorporate machine learning (ML) models into routine reaction planning to search large parameter spaces more efficiently than human intuition.

ML has shown great promise as a method for reaction planning and optimization, especially for expensive-to-evaluate problems. Bayesian optimization (BO) is particularly useful in this regard due to its exploration and exploitation policies, enabling rapid optimization with high precision even when applied to large and diverse search spaces.^2,7–10^ In BO, iterations of a probabilistic Gaussian process-based model are used to suggest input values in search of a global maximum, or minimum, in the reaction space.^11,12^ A response surface may be generated from the BO algorithm that interpolates and predicts further experiments within predefined parameter bounds.^6^ Shields *et al.* initially developed a Python package, experimental design *via* Bayesian optimization (EDBO), which has been demonstrated to be an effective tool for reaction planning and single-objective optimization.^2^ More recently, Garrido Torres *et al.* introduced EDBO+, a multi-objective active learning optimizer for chemical synthesis, which also includes updated features for modifying the reaction space mid-campaign, and improved data visualization methods.^6^ Multi-objective optimization enables simultaneous optimization of one or more reaction parameters (inputs), which in turn helps discover relationships between the objectives. Such methods have been proven effective in several cases, such as multi-step synthesis and continuous flow chemistry.^13–15^

In combination with Bayesian optimization, continuous flow synthesis techniques are powerful tools towards reaction optimization and the exploration of novel syntheses.^3,13,16–18^ Continuous flow chemistry offers a number of advantages including scalability and reproducibility as a result of automated liquid handling.^19^ These systems ensure that reagents flow at constant rates to maintain steady state conditions, and allow the reaction to run indefinitely if continuous manufacturing is desired.^20^ As a result of the high surface area-to-volume ratio of the millimeter size tubing, nearly instantaneous heat and mass transfer occurs, ensuring that reactions with hazardous intermediates can be safely controlled.^21–23^ When held under pressure, reactions may be conducted above the standard solvent boiling point, which readily allows access to an expanded reaction space. Additionally, the potential for in-line analytics (such as NMR, infrared spectroscopy (IR), *etc.*) and purifications or separations coupled with automation enhances the utility of flow techniques for high-throughput and autonomous experimentation.^24–31^ Recently, there have been tremendous strides made towards fully autonomous (closed-loop) experimentation systems that require little to no human intervention once initiated, and undoubtedly these systems will continue to mature and find value in research labs.^16,32–34^ In contrast to fully self-driving labs, there are many opportunities for human-in-the-loop and interactive ML to make an impact. Rather than being fully autonomous, these human/machine teams offer a data-driven approach with complementary human decision making and automated characterization steps in the workflow.^35,36^ These systems also have the inherent advantage of being straightforward to implement since they decrease the amount of software and hardware engineering needed, which can often be time intensive and costly. Furthermore, these workflows draw on the strengths of both the machine and human to perform interactive research.

While the methods described above have utility in many domain areas, one of the primary drivers has been active pharmaceutical ingredient research due to its market value. Further extension of these methods to functional material synthesis however, is desirable. Ionic groups provide unique material properties and have found wide utility in applications such as separations, adhesives, green synthetic solvents, and antibacterial agents (among many others) owing to their tunable structures, chemical resilience, thermal stability, and ease of processing.^37–43^ Ionic liquids (ILs) also have well documented utility in energy storage and conversion materials and devices.^44,45^ ILs and poly(ionic liquids) (PILs) are often comprised of cationic imidazolium or pyridinium salts, traditionally synthesized *via* a S_N_2 reaction of the starting nitrogen nucleophile with alkyl halides.^46^ One opportunity in IL synthesis is improving scalability since typical preparations are reported as benchtop batch reactions. By adapting the syntheses of these compounds to flow, ILs can be produced in larger quantities or on shorter timescales than those traditionally accessible in batch. Recently, Domański *et al.* described the acceleration of alkylimidazolium salt synthesis using a continuous flow and auto-frequency tuning microwave reactor platform.^47^ The application of microwaves enabled rapid product formation, with residence times under 10 minutes, yields approaching 97%, and production rates (PRs) on the order of several hundreds of grams per hour. Cao *et al.* also demonstrated a MW-assisted water-free flow synthesis of pyridinium salts on a similar timescale with >94% yield.^48^ These studies provided conditions with good conversions and yields, however, they both followed traditional small-scale optimization protocols varying one variable at a time (*i.e.* reaction time, residence time, or temperature). Furthermore, in an attempt to identify reaction trends using this method, the variable space is often purposely limited, which may hinder the search for global maxima (or minima). More recently, Pan *et al.* reported an advanced approach built on statistical design of experiments and active optimization for the purification of imidazolium ILs loaded with metal ions.^39^ This method identified global optimum conditions and demonstrated liquid–liquid extraction of ILs in continuous flow.

In the present study, we document the implementation of the multi-objective experimental design *via* Bayesian optimization (EDBO+) algorithm for human-in-the-loop optimization of the synthesis of butylpyridinium bromide under continuous flow.^6^ The use of EDBO+ in conjunction with flow chemistry served to reduce inconsistencies between reactions while enhancing scalability. The interactive loop helped identify a Pareto front, which represents a series of non-dominated solutions of the reaction outputs.^49^ In our system, this provides insight into the inherent tradeoff between yield and production rate. Impressively, the initial Pareto front was found in 30 experiments out of ∼10 000 possible discrete parameter combinations. We further demonstrate the versatility of EDBO+ to re-evaluate input data when the reaction space is altered during an optimization campaign *via* changes in the upper temperature limit. To examine EDBO+ models derived from data with different resolutions, we explore the model predictions based on quantitative low- and high-field ^1^H NMR spectra. Finally, we demonstrate our reaction substrate can be extended from butylpyridinium bromide, which exhibits ionic liquid character, to poly(4-vinylpyridine) (P4VP) for the synthesis of side-chain modified polymers using continuous flow.

## Results and discussion

### EDBO+ workflow and initial reaction campaign

**Scheme 1:**
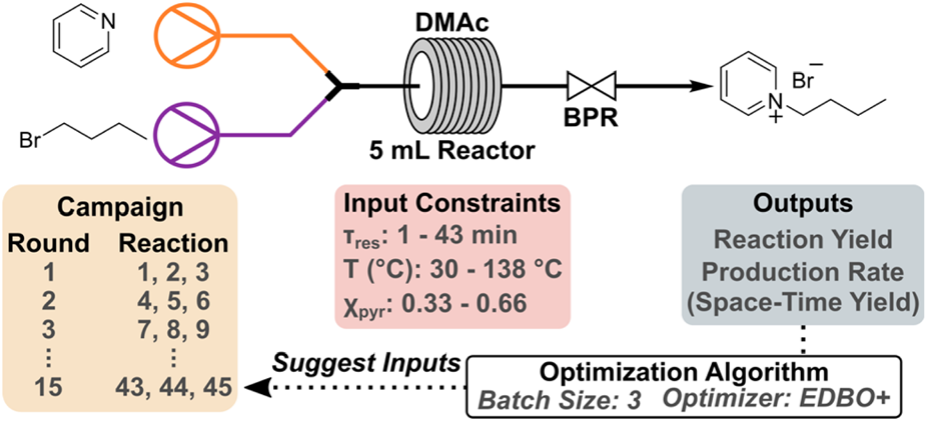
Continuous flow synthesis setup and EDBO+ workflow. Initial seed reactions were conducted within the predefined input constraints. Subsequent rounds of experiments were performed in batch sizes of three unique reactions. The outputs were used to update EDBO+ and provide the next round of suggested experiments. Initially 10 rounds of experiments were perform followed by expansion of the upper temperature constraint to 168 °C and another 5 rounds.

We employed the EDBO+ reaction planner developed by Garrido Torres *et al.* (which is also available as an open-source web application) to optimize the synthesis of butylpyridinium bromide under continuous flow, the workflow of which is outlined in Scheme 1.^6^ EDBO+ employs the Expected Hypervolume Improvement (q-EHVI) function which is designed to select a batch of points that jointly maximize the expected improvement over the current Pareto front. Additionally, we used the expected improvement (EI) function independently as a supplementary convergence criteria metric.^50–52^ The synthesis of butylpyridinium bromide was conducted in dimethylacetamide (DMAc) using a Vapourtec R-Series modular flow system. Pyridine and bromobutane (*n*-BuBr) were prepared as 1 M solutions in DMAc and subsequently combined *via* a mixer and flowed through a 5 mL perfluoroalkoxy (PFA) tube reactor. The flow rates of the two reagents were varied based on relative stoichiometry and time requirements. An aliquot of each reaction was collected while under steady state conditions, and then 1,3,5-trimethoxybenzene (TMB) was added as an internal standard for quantification *via*^1^H NMR spectroscopy. To launch the campaign, the reaction space was defined through three input parameters: residence time (*τ*_res_), temperature, and the mole fraction of pyridine (*χ*_pyr_). Initially, bounds on each input were established based on equipment limitations such that EDBO+ would not explore outside of the realm of possibility for the flow setup. For example, the temperature bounds could not exceed the safe operating limits of the flow reactor (150 °C for a standard PFA tube reactor). The residence time and temperature were constrained to 1–43 min and 30–138 °C, respectively, while the mole fraction of pyridine was kept between 0.33–0.66 (nominally 1 : 2–2 : 1 moles of pyridine relative to *n*-BuBr). The output for this campaign was set to simultaneously maximize the yield (%) and production rate (g h^−1^), the latter of which can be transformed to space-time yield (STY) (mmol mL^−1^ h^−1^) after taking into account the reactor volume. After conducting a set of three reactions suggested by EDBO+, the yield and production rate of product were calculated from quantitative ^1^H NMR experiments. Full details of the workflow for EDBO+ can be found in the ESI.†

To initiate EDBO+, four replicate reactions were conducted in the central region of each input range (23 min *τ*_res_, 85 °C, and 0.50 *χ*_pyr_) and used as seed reactions. These conditions were chosen to ensure an adequate output response while simultaneously providing insight into the reproducibility of the flow system workflow at the onset of the campaign. It should be noted that while the reaction campaign was initiated using conditions in the central region of the parameter space, the optimizer could have been initialized using other methods since past work has shown that these initialization methods converge over time.^6,53^ Overall, the conditions chosen to initialize the campaign provided an average yield of 15.03% (*σ* 1.74), production rate of 0.21 g h^−1^ (*σ* 0.02), and STY of 0.20 mmol mL^−1^ h^−1^ (*σ* 0.02) over the four data points confirming good reproducibility of the workflow. After manually inputting the results from the seed reaction and continuing the campaign, EDBO+ generated a predictive model and subsequently suggested new inputs within the upper and lower limits of the reaction space to test. The top three suggested experiments were then manually queued on the flow system and tested as an iteration (or round) of the reaction campaign and repeated until 10 rounds were complete.

**Fig. 1:**
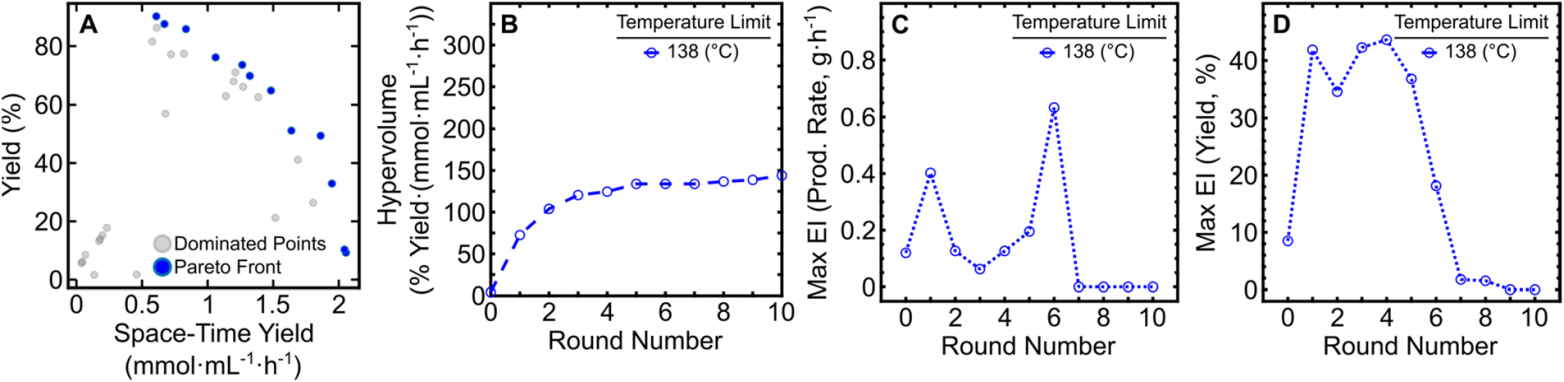
Monitoring metrics for the initial EDBO+ reaction optimization campaign. (A) The Pareto front solution of the multi-objective optimization (blue) and dominated solutions (grey). (B) Expansion of the hypervolume of all solutions to the Pareto front. (C) Maximum EI in production rate. (D) Maximum EI in reaction yield. Note that the EI for each round contains data from all previous experiments.

The resulting dataset from the 10-round campaign is comprised of dominated solutions (Fig. 1A, grey circles) and non-dominated solutions (Fig. 1A, blue circles) that form a Pareto front illustrating the tradeoff between product yield (%) and STY (mmol mL^−1^ h^−1^). As the campaign progressed, the front evolved over time as the algorithm attempted to increase the hypervolume of the Pareto front, defined as the area spanned by the front and a reference point in the two-dimensional space.^13^ By monitoring the change in hypervolume after each round of experiments, one may determine when to halt an optimization campaign (Fig. 1B). Qualitatively, the slope of the hypervolume represents the improvement in the Pareto front, since increases in slope represent expansion within the Pareto front. Large increases in hypervolume indicate identification of other non-dominated solutions and that further optimization is necessary. After the seventh round of the initial campaign, only marginal increases in the hypervolume were observed indicating minimal enhancements to the Pareto front. In addition, the maximum expected improvement (EI) in production rate (Fig. 1C) and reaction yield (Fig. 1D) reached a valley after round seven and maintained minor changes in EI through round 10. While round seven showed the lowest maximum EI values to that point, three additional rounds were required to ensure that the campaign reached a state of convergence. This provided a greater level of confidence in the optimization results, without lengthening the campaign dramatically. Considering changes in both the hypervolume of the Pareto front and EI in latter rounds, these results indicated that the campaign could be ended after round 10. It should be noted that because EDBO+ does not inherently identify one particular condition as optimal, the experimenter must still interpret the Pareto front to determine the “best condition” for their desired goal. Depending on the intended application, a low yield but high production rate (or *vice versa*) may be ideal. In our case, we found that moderately high yields (>80%) with production rates around 1 g h^−1^ best fit within the scope of this work to demonstrate the utility of EDBO+ for reaction optimization in flow. Our chosen “optimal” conditions for butylpyridinium bromide synthesis were determined to be at 138 °C, with a 21 min *τ*_res_ and 0.66 *χ*_pyr_, which had a yield of 85.86% and a production rate of 0.90 g h^−1^ (0.84 mmol mL^−1^ h^−1^ STY). One contributing factor in the selection of these conditions centered on the product being easy to purify, as evidenced by the 86% internal standard yield *versus* the 83% isolated yield. When paired with the low material cost of the reaction, this negated the need to push the reaction to a higher yield (>90%).

### Expansion of the reaction space to higher temperatures

During the 10-round campaign, we observed that the majority of suggested experiments tended to favor higher temperatures (namely 138 °C) as part of the EDBO+ exploration and exploitation policies. This is likely due to the high yields and moderate production rates achieved with mid-range residence times (see Table S1†). At this point, traditional closed-loop autonomous workflows would likely terminate the campaign due to campaign convergence. But our human-in-the-loop workflow helped identify that 19 of the 30 reactions had been conducted at 138 °C (the upper bound). While our initial reactions were limited to 138 °C because of the PFA tubing (which tends to be more affordable and is common in microfluidic setups), stainless steel tube reactors enable temperatures up to 250 °C. In an effort to expand the Pareto front, the upper temperature bound was in turn increased, and the reactor replaced with a 5 mL stainless steel tube reactor. Since higher temperatures may lead to reaction decomposition, a systematic temperature sweep of the optimal condition (21 min *τ*_res_ and 0.66 *χ*_pyr_) was first performed.

Upon manual elevation of the temperature from 138 °C to 160 °C, an improvement in the yield from 90% to ∼97% was observed (Fig. S8†), before plateauing between 160–170 °C. A similar trend was noted for production rates, with a maximum of 2.04 g h^−1^. At higher temperatures however, line broadening in the ^1^H NMR spectrum was observed (Fig. S9†) that signified reaction decomposition was starting to occur. This line broadening could lead to greater uncertainty in quantification and product purification challenges; therefore, the upper temperature limit for the reaction planner was set to 168 °C.

**Fig. 2:**
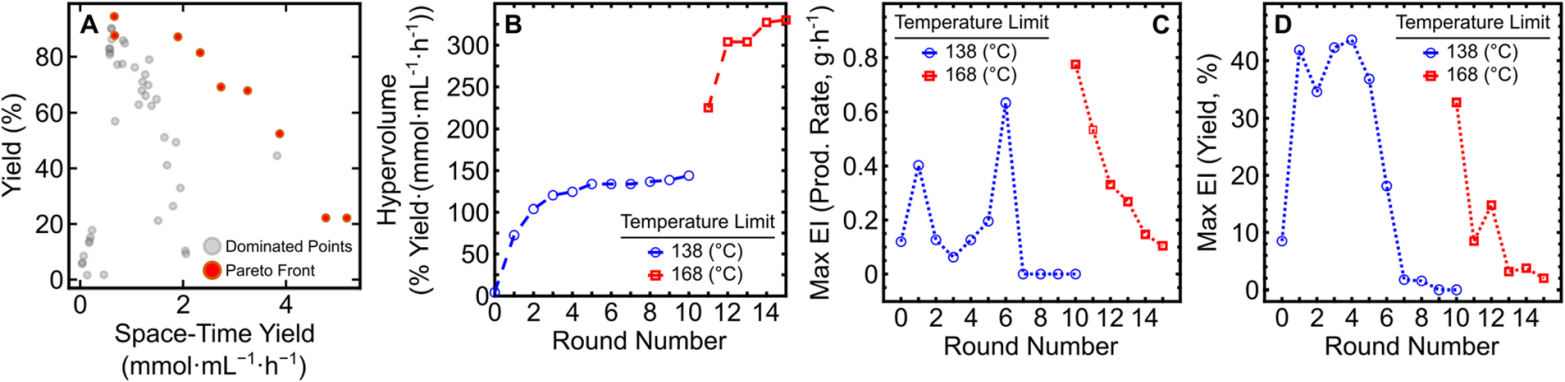
Monitoring metrics for the expanded EDBO+ reaction optimization campaign. (A) The Pareto front solution of the multi-objective optimization (red) and dominated solutions (grey). (B) Expansion of the hypervolume of all solutions to the Pareto front. (C) Maximum EI in production rate. (D) Maximum EI in reaction yield. The EI for each round contains data from all previous experiments. Data from the initial and expanded reaction campaigns are shown in blue and red, respectively.

With the expansion of the temperature bounds to 168 °C and concomitant increase in yield and production rate, a shift in the Pareto front occurred (Fig. 2A). By performing an additional five iterations of EDBO+ (using data from the pre-existing 10 round campaign) a production rate (PR) above 5 g h^−1^ could be obtained (PR: 5.60; STY: 5.18), as listed in Table 1. While higher production rates were obtained, the yields of those reactions were limited to under ∼50% due to insufficient reaction time. The Pareto front expansion corresponded to a large increase in hypervolume (Fig. 2B) and an initial increase in maximum EI for both target objectives. A steady reduction in the Pareto front expansion rate and maximum EI for the objectives could be seen over the five additional rounds (Fig. 2C and D). Underlying hyperparameter values of the variables in the surrogate models after round 10 and round 15 can be found in the ESI (Table S2 and S3).† These results highlight the versatility of EDBO+ to re-evaluate experimental datasets and perform further optimization when alterations are made to the reaction constraints mid-campaign.

**Table 1:** Experimental conditions for the highest yields and space-time yields achieved during the initial and expanded EDBO+ campaigns

Campaign/condition	Inputs			Outputs		
	Temperature (°C)	*τ*_res_ (min)	*χ*_pyr_	Yield (%)	Production rate (g h^−1^)	Space-time yield (mmol mL^−1^ h^−1^)
Initial (highest yield)	138	33	0.63	90.24	0.66	0.61
Initial (highest STY)	135	1	0.63	9.25	2.22	2.05
Expanded (highest yield)	156	29	0.66	94.48	0.72	0.66
Expanded (highest STY)	168	1	0.39	22.14	5.60	5.18
Optimal condition	138	21	0.66	85.86	0.90	0.84
Optimal (Isolated)^a^	138	21	0.66	82.97	0.87	0.81

a The product isolated from a reaction under the optimal conditions at 8× scale (8× the collection volume) was obtained as an off-white powder (1.22 g). Full details are provided in the ESI.

### EDBO+ predictions with low-resolution data

As flow synthesis techniques have become more popular, there has been a shift towards incorporating low-field analytics (such as NMR) either in-line, or on-line, with flow setups due to their lower cost and ease of use. While the higher signal-to-noise ratio achieved in high-field NMR is desirable—and often necessary for structural determination or two-dimensional experiments—recent improvements to low-field (60–100 MHz) NMR instruments have renewed interest for the flow chemistry community. Low-field NMR has several advantages over high-field NMR for coupling to flow setups, namely that they can be placed on the benchtop, utilize flow cells, and do not require the magnet to be cryogenically cooled. Solvent suppression negates the requirement for deuterated solvents, while continuous flow at steady state keeps product concentrations constant. Furthermore, low-field NMRs have proven to be effective tools for automated synthesis and reaction optimization under flow.^24,54–57^ Though benchtop NMR spectrometers are versatile for reaction monitoring, they remain limited due to poor resolution, especially where resonances are tightly distributed within the spectra, which leads to overlapping signals and greater uncertainty in quantification.^58,59^

To circumvent low-field NMR resolution limitations and reduce quantification errors, we relied on manual collection of 400 MHz NMR data to obtain reaction yields for the EDBO+ campaign presented above. However, understanding the role of low-resolution data on ML predictions is an important step towards more automated experimentation. Additionally, as automated flow setups coupled with computer-processed data gains popularity, it is important to compare the accuracy of these data analysis methods. To achieve this we employed nmrglue, available as an open-source Python module, for semi-autonomous processing of both 60 MHz and 400 MHz NMR spectra.^60^ In brief, raw ^1^H NMR data files were imported into nmrglue, followed by semi-automated phasing and baseline correction across the entire spectrum. The baseline was defined through manual selection to prevent nmrglue from selecting erroneous points along the *x*-axis. Peaks of interest were integrated within predefined integration windows and calibrated based on the internal standard (TMB) singlet at 5.2 ppm (3H).

The results for the reaction yields, production rates, and STYs determined from manual and semi-automated processing on low- and high-field NMR are summarized in Table S4, Fig. S11 and S12.† We determined the mean absolute error (MAE) in STY and yield to compare the relative accuracies of each analysis and data acquisition method (Table S5†). Since manual phasing and integration of NMR data is more common in reaction optimizations, we accept the manually processed 400 MHz data used in the campaign as ground truth (0.0 MAE). Of the other three methods, the most accurate analysis came from yields calculated from 400 MHz data *via* nmrglue (2.9 MAE). The 60 MHz data proved least accurate relative to the high-resolution analogues, with 4.4 and 8.0 MAE for semi-automated and manually processed yields, respectively.

**Fig. 3:**
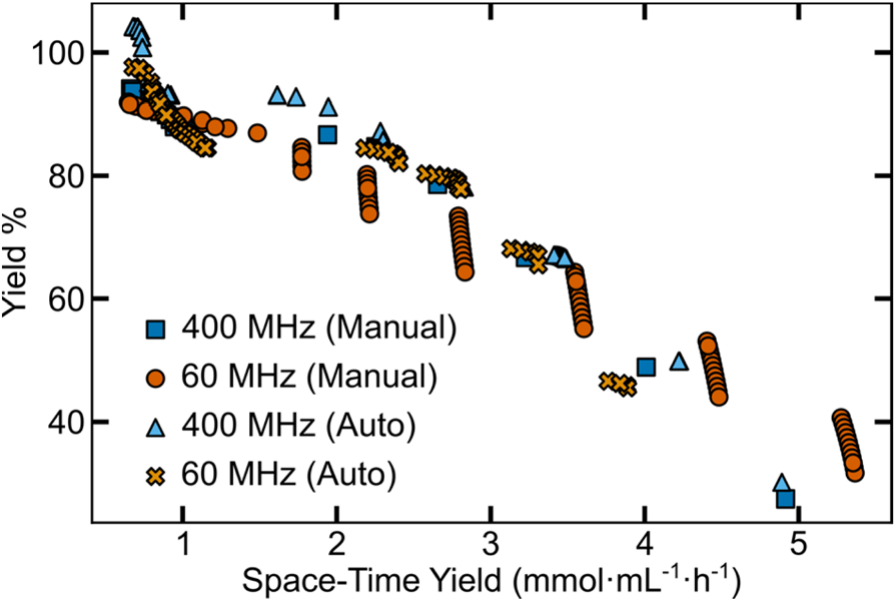
Predicted Pareto fronts from low- and high-field NMR analysis outputs of manually and semi-automated processed data.

To determine the effect of these discrepancies on EDBO+, we generated predictions from separate input files and calculated the predicted Pareto front for the expanded EDBO+ campaign. The predicted Pareto fronts shown in Fig. 3 were obtained by incorporating the input data from each of the four analysis methods for the 15-round campaign into the Gaussian process regression (GPR) model of EDBO+, and generating predictions for the entire dataset (∼10 000 experimental conditions). The predicted Pareto fronts, including uncertainties in the predicted outputs from the BO model, are depicted in Fig. S14 and S15.†

Although similar in shape, the Pareto fronts predicted from 60 MHz data had noticeably larger uncertainty values. In contrast, the 400 MHz predictions for both manually- and nmrglue-processed outputs are most similar, as shown in Fig. S14.† Predictions built from the 400 MHz data also closely match the experimental Pareto front (Fig. 2A) from the reaction campaign. To further quantify the similarity of the predictions, we extracted the hypervolume of the predicted Pareto fronts (Table S6†). The manually processed 400 MHz and 60 MHz NMR data had hypervolumes of 334 and 370% yield mmol mL^−1^ h^−1^ respectively, while semi-automated processing tended to reach lower values of 308 and 363% yield mmol mL^−1^ h^−1^ for the 400 MHz and 60 MHz data, respectively. Compared to the hypervolume from the experimental data (330% yield mmol mL^−1^ h^−1^), the 400 MHz predictions were a closer match to the experimental Pareto front (Fig. 2A) than the 60 MHz predictions. It is worth noting that after 15 rounds (45 experiments) the maximum difference between the experimental and predicted hypervolume is ∼12% which may (or may not) be acceptable for a given reaction optimization. While this analysis provides some insight into role of analysis methods on model predictions, it is likely that the experimental points the EDBO+ workflow suggests to arrive at the Pareto front would be different if run as independent campaigns.

These results indicated that EDBO+ is able to provide reasonable predictions from low- or high-field NMR data, albeit at higher uncertainty levels. Future research exploring these effects on optimization algorithms is ongoing since there are instances when compromises must be made between autonomous workflows and high fidelity characterization.

### Application of the reaction conditions to a representative polymer

Compared to polymeric materials, the characterization of small molecule reactions offer a number of advantages that stem from well-established solution state high-throughput characterization techniques (high performance liquid chromatography (HPLC), NMR, mass spectrometry (MS), *etc.*). To test whether knowledge gained from small molecule surrogate reactions can be readily transferred to polymeric systems, we extended the substrate scope to a representative polymer, poly(4-vinylpyridine) (P4VP), which served as the substrate for quaternization by bromobutane. We hypothesized that P4VP should serve as an excellent nucleophile for quaternization due to its abundance of pyridine moieties along the polymer chain, and compatibility with DMAc.

The quaternized product, poly[(4-vinylpyridine)-*co*-(*N*-butylpyridinium bromide)], (*f*-P4VP), was prepared following the procedures outlined in the ESI.† We initially attempted to functionalize P4VP under the user-defined optimal reaction conditions on the Pareto front (138 °C, with a 21 min *τ*_res_ and 0.66 *χ*_pyr_); however, precipitation of the polymer within the reactor upon quaternization occurred due to high degrees of functionalization. Therefore, to avoid precipitation of the polymer at high temperatures and long residence times, conditions were selected from the EDBO+ reaction campaign Pareto front such that an effective quaternization of ∼10% would be achieved. In brief, a solution of P4VP was prepared in DMAc with a concentration of 1 M pyridine and reacted with 1 M bromobutane in DMAc (Scheme S1†) for 1 min *τ*_res_ at 135 °C, and with 0.63 *χ*_pyr_. The product was collected and purified through precipitation, then dried on a Schlenk line as a white powder for further analysis.

**Fig. 4:**
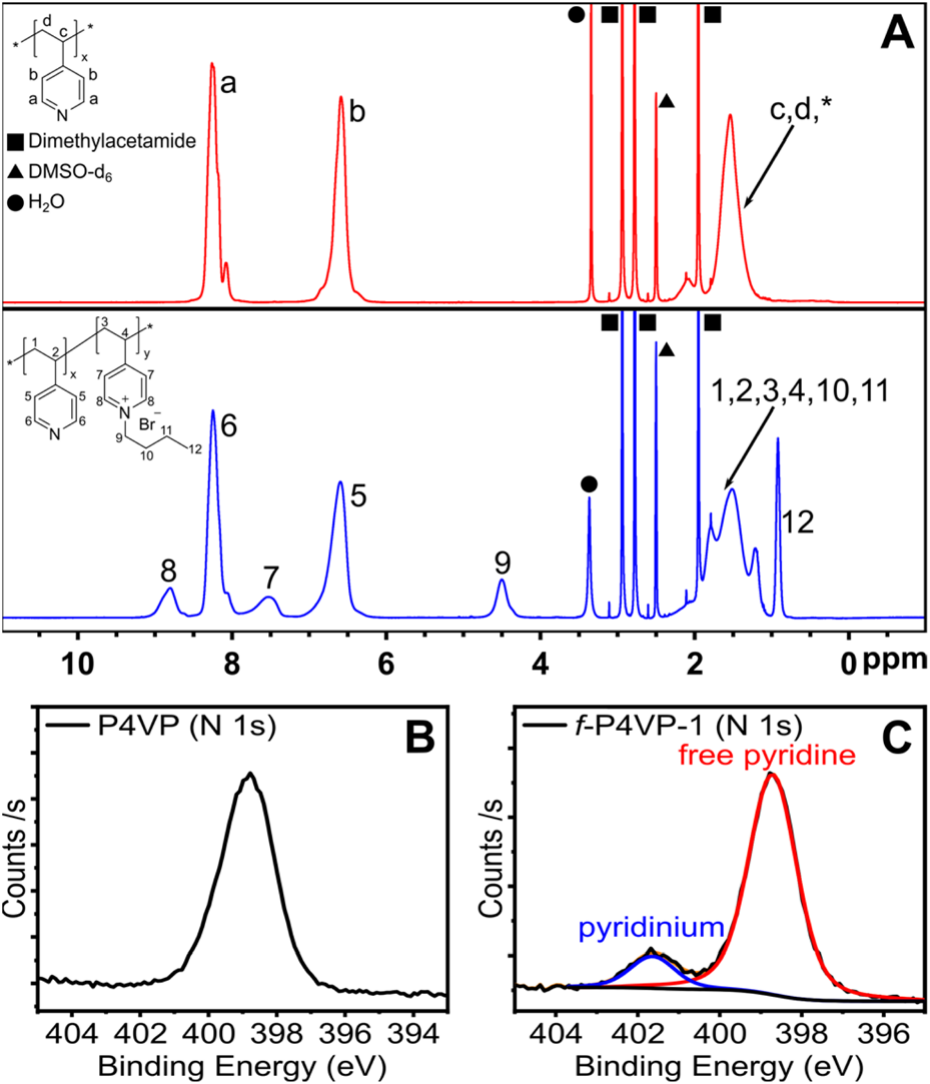
Characterization of the polymer product synthesized under continuous flow. (A) ^1^H NMR spectra of P4VP (top, red) and *f*-P4VP-1 (bottom, blue) in DMSO-d_6_. (B). XPS N 1s spectrum of P4VP. (C). XPS N 1s spectrum of *f*-P4VP-1. Analysis reveals two distinct N species at 398.71 eV and 401.64 eV, corresponding to free pyridine and quaternized pyridinium on the polymer, respectively. Samples were isolated from solutions in DMAc prior to NMR and XPS analysis.

We set out to confirm quaternization of the P4VP and directly compare conversion to the small-molecule surrogate reaction of free pyridine. To confirm reaction conversion, we employed ^1^H NMR and X-ray photoelectron spectroscopy (XPS) as shown in Fig. 4. Comparing the ^1^H NMR spectrum of un-functionalized P4VP to *f*-P4VP-1, we first identified the appearance of a broad resonance at 4.5 ppm from the butyl carbon alpha to the pyridinium. Persistence of this peak after purification indicated that polymer functionalization had occurred. We also observed two broad peaks at ∼7.5 and ∼8.8 ppm resulting from pyridinium groups on the modified polymer and used XPS to quantify the degree of functionalization. We observed two species of nitrogen in the N 1s spectrum of the quaternized product *f*-P4VP-1 (Fig. 4C), while only pyridine was detected in P4VP (Fig. 4B). In the *f*-P4VP-1 sample, the large peak at 398.7 eV corresponds to unmodified pyridine functional groups in the polymer, while the peak at higher binding energy (401.6 eV) corresponds to pyridinium groups. Peak fitting of the two regions showed 12% quaternization in *f*-P4VP-1, which was slightly higher than the yield of butylpyridinium bromide synthesized under identical conditions (9.25%, see Table 1). Furthermore, XPS survey spectra of P4VP and the functionalized product (Fig. S18†) revealed the introduction of bromine after quaternization.

To provide evidence that the reaction caused a change in the material properties of P4VP, we performed thermal analysis by differential scanning calorimetry (DSC) and thermogravimetric analysis (TGA) (Fig. S17†). TGA of the *f*-P4VP-1 under an inert atmosphere revealed a decrease in thermal stability upon quaternization relative to unmodified P4VP. For both polymers, an initial decrease in mass upon an isothermal hold at 100 °C occurred due to the loss of adsorbed water or residual solvent, which was also observed in the first heat cycle of DSC. The major degradation event occurred at ∼275–400 °C for *f*-P4VP-1 and ∼350–450 °C for P4VP respectively, which aligns with the prior report of iodomethane- based quaternization of P4VP reported by Mavronasou *et al.*^61^ This decrease in thermal stability can be attributed to Hofmann elimination reactions due to the ammonium groups at high temperatures.^62^ Additional experiments were also performed to further compare the chemical reactivity of poly(4-vinylpyridine) and free pyridine under various degrees of functionalization. To limit flow reaction incompatibilities due to precipitation of functionalized polymer, these reactions were done using batch chemistry. To directly compare reactivity, we performed three extra reactions using previously tested reaction conditions from the small molecule EDBO+ campaign. Additionally, one reaction condition was also selected that had not been previously tested to compare the EDBO+ yield predictions to polymer functionalization. While the lower yield reaction conditions (under ∼15%) provided a soluble reaction mixture, the other three conditions (above ∼60%) all very quickly led to precipitate in the reaction mixture. This illustrates that considerations beyond merely chemical reactivity must be made when extending small molecule datasets to polymer functionalization. After isolating the polymer products, ^1^H NMR spectroscopy and XPS were performed to determine the percent of functionalization (Table S7†). These results pointed to good correlation between small molecule and polymer reactivity, illustrating the value of the small molecule dataset. At high conversion, we observed some deviation between polymer-bound pyridine and small molecule pyridine reactivity. At these conditions, the small molecule pyridine provided 90% yield *via*^1^H-NMR while the poly(4-vinylpyridine) gave 76% atomic conversion *via* XPS. This is likely a result of the steric effects of the ionic groups present on the polymer backbone at high functionalization. To further illustrate ionic effects on the material we acquired TGA and DSC of these *f*-P4VP samples. The DSC traces provided additional support that upon increasing the functionalization of the pyridine side-chain the structures become progressively more rigid, limiting free polymer mobility. We observed an increase of *T*_g_ from 141 °C to 174 °C upon 15% functionalization. Above 60% functionalization the *T*_g_ cannot be observed *via* DSC within the temperature window due to polymer rigidity, which is consistent with previous reports.^61^ TGA also confirmed that all quaternized polymers were less thermally stable than unfunctionalized P4VP. This was consistent with our initial observation that functionalized polymers lose approximately 5–10 wt% mass as a result of residual water and then at temperatures of 275–400 °C the material undergoes degradation. Overall, the expansion of our reaction conditions from the small molecule EDBO+ campaign to P4VP functionalization demonstrated the utility of our flow setup and showed that small molecules may be used as surrogate reactions for polymeric systems (or indeed other complex systems), with aid from ML and active learning.

## Conclusions

This work demonstrated the application of a human-in-the-loop multi-objective Bayesian optimization platform (EDBO+) towards the production of butylpyridinium bromide under continuous flow conditions. The EDBO+ algorithm was implemented to simultaneously optimize the reaction yield and production rate (or STY) of the product, and assist in reaction planning by suggesting new experimental inputs of reaction stoichiometry, residence time, and temperature. After only 30 experiments, out of ∼10 000 possible discrete input parameter combinations, a well-defined Pareto front provided insight into the trade-off between outputs. Furthermore, as the reaction campaign evolved, our human-in-the-loop design allowed for additional questions to be asked, and knowledge to be gained. In an attempt to push the Pareto front to previously inaccessible regions, the permitted temperature was increased and the planner was able to quickly re-optimize the objectives.

Due to the increasing interest in low-field analytics and automated data processing, we sought to compare the accuracy of outputs obtained from manually and semi-automated processing of high-field (400 MHz) and low-field (60 MHz) NMR spectrometers. Results indicate that semi-automated processing of low-field NMR spectra for data analysis can be effective, however, high-field data is preferred. We further analysed the resilience of EDBO+ predictions when 60 MHz data was used instead of 400 MHz data. Based on predictions of the Pareto front and hypervolume, the semi-automated 400 MHz data predictions closely matched experimental data from the reaction campaign. Even when the EDBO+ model was trained on low fidelity data, the hypervolume of the predicted Pareto front only displayed a 12% difference when compared to the experimental data. These studies provide insight on the role of data acquisition and processing in surrogate machine learning algorithms.

The combination of human-in-the-loop interactive machine learning research coupled with continuous flow chemistry presents a powerful tool for chemical synthesis and reaction optimization. Furthermore, these results point to the utility of small molecule surrogate reactions and extension of these methods to functional materials synthesis.

## Data availability

Experimental conditions and characterization are provided in the ESI.† Datasets and a Python-based notebook supporting this article have also been uploaded as part of the ESI.†

## Author contributions

Conceptualization: L. A. B., C. A. C.; data curation: J. H. D., J. G. E., L. A. B.; formal analysis: J. H. D., J. G. E., A. A. N., P. M., L. A. B.; funding acquisition: T. M. S., R. A. V., C. A. C., L. A. B.; investigation: J. H. D., J. G. E., A. A. N., S. I., S. L. L.,H. F.; methodology: J. H. D., J. G. E., L. A. B.; project administration: L. A. B., C. A. C.; resources: J. A. G. T., A. G. D., T. M. S., L. A. B; software: J. A. G. T., A. G. D., J. G. E., S. I., P. M., L. A. B.; supervision: A. G. D., T. M. S., R. A. V., C. A. C., L. A. B.; validation: J. H. D., J. G. E., L. A. B.; visualization: J. H. D., J. G. E., A. A. N., S. I., S. L. L., H. F., L. A. B.; writing – original draft: J. H. D.; writing – review & editing: J. H. D., J. G. E., A. A. N, R. A. V., C. A. C., L. A. B.

## Conflicts of interest

There are no conflicts of interest to declare.

## Supplementary Material

SC-014-D3SC01303K-s001

SC-014-D3SC01303K-s002
