# Supplementary material for: Continuous flow synthesis of pyridinium salts accelerated by multi-objective Bayesian optimization with active learning
Source: Chem Sci. 2023 Jul 12;14(30):8061–9. doi: 10.1039/d3sc01303k (PMC10395269; doi:10.1039/d3sc01303k)
Supplement: SC-014-D3SC01303K-s001 [file SC-014-D3SC01303K-s001.pdf]

Electronic Supplementary Material (ESI) for Chemical Science.
This journal is © The Royal Society of Chemistry 2023

Supporting Information (SI) for:

Continuous Flow Synthesis of Pyridinium Salts Accelerated by Multi-Objective Bayesian Optimization with Active Learning

John H. Dunlap[a,b], Jeffrey G. Ethier[a,b], Amelia A. Putnam-Neeb[a,c], Sanjay Iyer[d], Shao-Xiong Lennon Luo[e], Haosheng Feng[e], Jose Antonio Garrido Torres[f], Abigail G. Doyle[g], Timothy M. Swager[e], Richard A. Vaia[a], Peter Mirau[a], Christopher A. Crouse[a], Luke A. Baldwin[a,*]

[a]Materials and Manufacturing Directorate, Air Force Research Laboratory, Wright-Patterson AFB, OH, 45433, USA
[b]UES, Inc., Dayton, OH, 45431, USA
[c]National Research Council Research Associate, Air Force Research Laboratory, Wright-Patterson AFB, OH, 45433, USA
[d]Department of Chemistry, Purdue University, West Lafayette, IN, 47907, USA
[e]Department of Chemistry, Massachusetts Institute of Technology, Cambridge, MA, 02139, USA
[f]Department of Chemistry, Princeton University, Princeton, NJ, 08544, USA
[g]Department of Chemistry and Biochemistry, University of California, Los Angeles, CA, 90095, USA

[*]Corresponding Author: luke.baldwin.1@us.af.mil

Contents *Page*

Materials. S2

Optimization Details. S2-3

Experimental Details. S3-6

Scheme S1. Continuous Flow Chemistry Setup for *f*-P4VP Synthesis. S6

Figure S1-S3. ^{1}H NMR of Butylpyridinium Bromide and Reagents. S7-8

Figure S4-6. ^{1}H NMR of Isolated Butylpyridinium Bromide. S8-9

Figure S7. ^{13}C NMR of Isolated Butylpyridinium Bromide. S10

Figure S8. Reaction Yield as a Function of Temperature. S10

Figure S9. ^{1}H NMR of Butylpyridinium Bromide as a Function of Temperature. S11

Figure S10. ^{1}H NMR of Butylpyridinium Bromide Measured on a 400 MHz and 60 MHz NMR. S11

Table S1. Input Parameters and Output Values for the EDBO+ Reaction Campaign. S12

Table S2. Hyperparameter Values of the Surrogate Model Variables after Round 10. S13

Table S3. Hyperparameter Values of the Surrogate Model Variables after Round 15. S13

Table S4. Comparison of Output Values Calculated From Low- and High-Field NMR Data. S14

Table S5. Error in NMR Outputs Determined from Low- and High- Field NMR Data Analysis. S15

Figure S11. Space-Time Yields Calculated from Manual and Automated Processing Methods. S15

Figure S12. Yields Calculated from Manual and Automated Processing Methods. S15

Figure S13. Boxplot of NMR Data from 60 and 400 MHz NMR Integrations. S16

Figure S14. Predicted Pareto Fronts and Dominated Predictions for all Analytical Methods. S16

Figure S15. Predicted Pareto Fronts for all Analytical Methods. S17

Table S6. Predicted Maximum Hypervolume of the Pareto Front After Round 15. S17

Figure S16. ^{13}C NMR of Isolated Polymer Product (*f*-P4VP) and Poly(4-vinylpyridine). S18

Figure S17. Thermal Analysis of Polymer Samples. S18

Figure S18. X-ray Photoelectron Spectroscopy of Polymer Samples. S19

Figure S19. X-ray Photoelectron Spectroscopy of *f*-P4VP Samples Prepared in Batch. S19

Figure S20. Thermogravimetric Analysis (TGA) of *f*-P4VP Samples Prepared in Batch. S20

Figure S21. Differential Scanning Calorimetry (DSC) of f-P4VP Samples Prepared in Batch. S20

Table S7. Quaternization Yields of *f*-P4VP Prepared in Batch by ^{1}H NMR and XPS Analysis. S21

References. S21

Materials.
All chemicals were used as received without further purification unless otherwise indicated. Dimethyl sulfoxide-d_6 (DMSO-d_6, 99.5 %) was purchased from Alfa Aesar. 2-propanol (IPA, 99.5 %) and ethyl acetate (EtOAc, 99.5%) were purchased from Fisher Scientific. Pyridine (anhydrous, 99.5+ %) was purchased from Thermo Scientific. Hexane (99.9+ %) was purchased from Burdick & Jackson. Poly(4-vinylpyridine) (P4VP, 200k Da) was purchased from Scientific Polymer Products, Inc. 1-Bromobutane (*n*-BuBr, 99%), 1,3,5-trimethoxybenzene (TMB, 99+ %), N,N-dimethylacetamide (DMAc, anhydrous, 99.8 %), diethyl ether (inhibitor-free, 99.9+ %), and chloroform-d ($CDCl_3$, 99.8%) were purchased from Sigma Aldrich.

Optimization Details.

Experimental Design via Bayesian Optimization (EDBO+) Workflow.
The multi-objective optimization platform used is the open-source Experimental Design via Bayesian Optimization (EDBO+) toolkit. Details of the workflow can be found in references 4 and 5 of the main manuscript, but are briefly summarized here.[1,2] First, a reaction parameter space was defined (*e.g.*, temperature, residence time, *etc.*) to search during the optimization campaign. Our parameter space was chosen such that ~10,000 unique reaction conditions were searched, with temperatures ranging between 30-138 °C (or 30-168 °C), residence times between 1-43 min, and pyridine mole fraction between 0.33-

0.66. The optimization objectives were the yield and production rate of the butylpyridinium bromide product. While EDBO+ can suggest an initial set of experimental conditions, the center point of the parameter space was selected instead. The outputs of the initial experiments (*i.e.*, yield and production rate) were input to the EDBO+ platform and a set of new experimental conditions were suggested. The batch size for each round of suggestions was three experiments, and rounds were repeated until the hypervolume of the Pareto front no longer increased.

Relevant code used to initiate the reaction scope, run the campaign, define Pareto front and calculate the hypervolume are included in separate .ipynb file in supporting info. Hypervolume was calculated using pymoo: Multi-objective Optimization in Python (https://pymoo.org/) which is maintained by Julian Blank as an open source Python library.

The active learning algorithm implemented in the EDBO+ platform combines a Gaussian Process regression (GPR) model with the q-Expected HyperVolume Improvement (q-EHVI) acquisition function for multi-objective optimization. First, the model is trained on experimental data, and then predicts the outputs for the remaining untested reaction conditions in the parameter space. The acquisition function then ranks the untested experimental conditions based on the model predictions and uncertainties. The next set of experimental conditions are suggested from this ranking and the subsequent experiments are conducted at each iteration (round) of the campaign. With additional rounds, the accuracy of the GPR surrogate model increases and therefore the model is used to show predictions of the yield and production rate from all reaction conditions in Figure 3 of the manuscript. The hyperparameters of the GPR model after training on 10 experimental rounds as well as the final round are listed in Table S2 and S3, respectively. Since the automatic relevance determination (ARD) method is implemented in EDBO+, the hyperparameters listed are the characteristic length scales of the Matérn kernel for each input variable. Larger values indicate that the covariance is independent of the input variable, and therefore has less relevance to the output prediction.

Experimental Details.

Flow Reactor Setup.
Butylpyridinium bromide was synthesized using a Vapourtec R-Series modular flow chemistry system equipped with R2 C+ piston pumps and R2 S+ peristaltic pumps. Reactions were conducted in 5 mL tube reactors, either made of PFA (< 140 °C) or stainless steel (> 140 °C). A back pressure regulator (BPR) was used to increase the system pressure to prevent the solvent from boiling at high temperatures. Reagents were combined in a Y-mixer prior to entering the reactor. An 8 bar BPR was used in the case of reactions under 140 °C, while those at higher temperatures required a 17 bar BPR. Aliquots of product solutions were collected under steady state conditions via a Gilson GX-241 Fraction Collector.

Butylpyridinium Bromide Synthesis.
In brief, 1.0 M solutions of pyridine and bromobutane were prepared in dimethylacetamide (DMAc). Prior to loading the flow system, the tubing, pumps, mixer, BPR, and reactor were thoroughly rinsed with 2-propanol (IPA) then conditioned with DMAc. The reaction temperatures, residence times (τ_{res}), and stoichiometries were determined based on EDBO+ AI Planner inputs within the predetermined parameter bounds. The flow rate (mL/min) for each reaction varied based on the τ_{res} and mole fraction of pyridine and bromobutane. A 2.5 mL aliquot of the product solution was collected for further analysis under steady state conditions. Steady state was calculated by Vapourtec's Flow Commander software.

Butylpyridinium Bromide Isolation.
To test reaction scalability, butylpyridinium bromide was prepared at 8x scale (8x the collection volume of the EDBO+ campaign reactions) and isolated from the crude product mixture as follows. First, the product was transferred to centrifuge tubes and ethyl acetate (EtOAc) added in a 2:1 v/v EtOAc/DMAc ratio, then stored in a -5 °C freezer for 2 hours to precipitate the product. Immediately after chilling, the solutions were

centrifuged (927 x *g* /5 min). The supernatant was decanted, and the product washed with hexane and centrifuged three more times. Finally, the isolated product was dried under vacuum on a Schleck line and stored in an N_2 purge box. The product was isolated as an off-white powder (1.22 g, 83%). ^{1}H NMR (400 MHz, $CDCl_3$) δ (ppm) 9.58 (d, 2H), 8.52 (t, 1H), 8.13 (t, 2H), 4.98 (t, 2H), 2.00 (m, 2H), 1.38 (m, 2H), 0.91 (t, 3H). ^{13}C (100 MHz, $CDCl_3$) δ (ppm) 145.29, 145.21, 128.53, 61.75, 33.92, 19.35, 13.59.

The melting point was obtained using a Buchi M-565 melting point system from three aliquots of an isolated sample, with an average of 102 °C. It should be noted that as an ionic salt, butylpyridinium bromide is hygroscopic, and sufficiently dry samples were precipitated with EtOAc as solids. However, those that had absorbed water initially formed biphasic mixtures with EtOAc addition, though they were finally isolated as solids once dried under vacuum.

Flow synthesis of poly[(4-vinylpyridine)-*co*-(N-butylpyridinium bromide)] Synthesis.
The synthesis of poly[(4-vinylpyridine)-*co*-(N-butylpyridinium bromide)] (*f*-P4VP-1) was conducted in continuous flow by the reaction between P4VP and *n*-BuBr in a 2 mL PFA tube reactor. Due to solubility concerns at high concentrations and longer residence times where high degrees of functionalization may be reached, a reaction condition was chosen such that nominally < 10% quaternization of the pyridine moiety would be achieved. Solutions of 1 M *n*-BuBr and P4VP were prepared in DMAc and reacted at 135 °C for 1 min τ_{res}, with a pyridine mole fraction of 0.63 χ_{Pyr}. To prepare P4VP in DMAc at this concentration, the solution was heated to 45 °C and stirred until all solid dissolved, then diluted to volume in a volumetric flask. It should be noted that the concentration of P4VP was based on the quantity of pyridine in the polymer (i.e. 1M in [Pyridine]). The amount of pyridine in the P4VP was determined to be 0.0171 moles pyridine per g P4VP based on quantitative ^{1}H NMR measurements. Functionalization results can been seen in **Table S7**.

Batch Synthesis of poly[(4-vinylpyridine)-*co*-(N-butylpyridinium bromide)] Synthesis.
To explore reactivity and determine if *f*-P4VP would precipitate as the degree of functionalization increased, batch experiments were performed on the benchtop. In brief, 1 M solutions of P4VP and *n*-BuBr in DMAc were prepared, and mixed in glass vials according to the mole ratios described below, then heated with stirring on a hotplate at the corresponding temperature. Three reaction conditions that were previously explored during the small molecule EDBO+ campaign were selected (*f*-P4VP-2, -3, and -4), as well as a previously untested set of conditions (*f*-P4VP-5). Conditions with times greater than 15 minutes allowed for thorough and even heating of the samples. After the reaction had run for the allotted time, the samples were cooled to room temperature and immediately isolated by precipitation with diethyl ether as described in the following section. Precipitation occurred in *f*-P4VP-3**,** *f*-P4VP-4**,** and *f*-P4VP-5 due to product insolubility at high conversions, indicating that reactions with higher yields (> 60%) would be incompatible with continuous flow. The reaction conditions are listed below, and the yields provided in **Table S7**.

f-P4VP-2: 85 °C, 23 min, 0.50 χ_{Pyr}
f-P4VP-3: 138 °C, 23 min, 0.41 χ_{Pyr}
f-P4VP-4: 138 °C, 33 min, 0.63 χ_{Pyr}
f-P4VP-5: 123 °C, 23 min, 0.42 χ_{Pyr}

Poly[(4-vinylpyridine)-*co*-(N-butylpyridinium bromide)] Isolation.
To test reaction scalability and produce a larger amount of product, Poly[(4-vinylpyridine)-*co*-(N-butylpyridinium bromide)] (*f*-P4VP-1)was prepared at 4x scale and isolated from the crude product mixture. The crude product was precipitated from DMAc with diethyl ether (~1:1 v/v DMAc/ether) and centrifuged (2576 x *g* /5 min). The supernatant was then decanted to waste, and the product washed with diethyl ether three more times. The final collected precipitate was transferred to a Schlenk flask, dried under vacuum, and stored in an N_2 purge box. The product was isolated as a white powder (890 mg). For ^{1}H and ^{13}C NMR, an aliquot of the isolated polymer was dissolved in DMSO-d_6. ^{1}H NMR (400 MHz, $(CD_3)_2SO$) δ (ppm)

8.81 (br d, 2H), 8.25 (br d, 2H), 7.53 (br d, 2H), 6.60 (br d, 2H), 4.50 (br t, 2H), 2.39-1.05 (br m, 10H), 0.92 (br t, 3H). ^{13}C NMR (100 MHz, $(CD_3)_2SO$) δ (ppm) 153.06, 152.39, 149.44, 143.94, 126.89, 122.82, 59.62, 32.58, 18.75, 13.30.

NMR Characterization of Reagents and Products.
^{1}H NMR measurements of the reagents and products were collected on a Bruker AVANCE 400 MHz spectrometer. For comparison, spectra were also obtained using a Nanalysis NMReady-60pro 60 MHz benchtop spectrometer. For quantitative measurements 150 µL of 2M 1,3,5-trimethoxybenzene (TMB) in DMAc was added directly to the collected product, an aliquot of which was then diluted in chloroform-d ($CDCl_3$) to form a 1:1 $CDCl_3$/DMAc v/v solution.

Quantification by ^{1}H NMR Integration.
Manual Processing: The reaction yield and production rate for each reaction was calculated from quantitative ^{1}H NMR measurements of the crude product based on the theoretical yield. The theoretical yields (n_{Theo}, **Equation 1**) were calculated from the collection volume (V_{coll}, mL), reagent flow rates (mL/min), and limiting reagent (LR) concentrations (M).

$$n_{Theo} = \frac{V_{coll}}{Total\ Flow\ Rate} \times \frac{Flow\ Rate_{LR}\ [LR]}{1000} \tag{1}$$

The moles of product (n_{Pdt}) were obtained by the relative ratios of ^{1}H NMR integrations between the product and internal standard, calibrated to the TMB singlet peak (5.15 ppm, 3 H). The farthest downfield resonance of the product (d, 8.75 ppm, 2H), or the triplet at 7.77 ppm (1H), were used to calculate n_{Pdt} (**Equation 2**). Finally, the Yield (%) was determined via **Equation 3**.

$$n_{Pdt} = n_{TMB} \times \frac{3}{\int TMB} \times \frac{\int Pdt}{2} \tag{2}$$

$$\%\ Yield = \frac{n_{Pdt}}{n_{Theo}} \times 100 \tag{3}$$

Separately, the production rate (g·h^{-1}) may be determined by considering the reactor volume ($V_{reactor}$, mL), collection volume (V_{coll}, mL), residence time (τ_{res}, min), and the mass of product (m_{Pdt}) obtained as shown in **Equation 4**. Alternatively, the production rate may be expressed via Space-Time Yield (STY, mmol·mL^{-1}·h^{-1}), which allows for comparison between reactors of different volumes (**Equation 5**). In this study, the reactor size was 5 mL for all EDBO+ experiments. Note that the moles of product is the amount of product per one reactor volume.

$$Production\ Rate = \frac{m_{Pdt}\left(\frac{V_{reactor}}{V_{coll}}\right)}{\frac{\tau_{res}}{60}} \tag{4}$$

$$STY = \frac{(n_{Pdt})}{\frac{\tau_{res}}{60}(V_{reactor})} \tag{5}$$

A summary of the yields and production rates for each reaction within the EDBO+ campaign are provided in **Table S1** below.

Automatic processing: For samples using semi-automated processing, rather than manual, the data obtained from the 60 MHz and 400 MHz NMR instruments was processed using a python script to convert the raw data to an Excel table of integrated intensities from which the yields were calculated. Because of data imperfections and the presence of large solvent peaks (resulting from DMAc) the data was multiplied using a 1 Hz line broadening, Fourier transformed, automatically phased, then baseline corrected before peak integration. Processing of NMR data made use of standard Python libraries and the NMRGLUE library for

reading the data and some processing functions (https://www.nmrglue.com/).[3] The automatic baseline correction used selected points from the baseline with no signal and an integration width of 0.3 ppm. Peak integrals were calibrated based on the internal standard (TMB) peak at ~5.15 ppm (s, 3H). For butylpyridinium bromide quantification, the farthest downfield peak at ~8.8 ppm (d, 2H) was used. We observed peak overlap of this doublet with free or unreacted pyridine resonances in some samples that were synthesized at higher reaction temperatures, which would have led to inaccurate integration values. To accurately quantify these products, the triplet at ~7.8 ppm corresponding to the product (t, 1H) was used, and the calculation for yield was adjusted accordingly. We note that as a result of the DMAc/$CDCl_3$ solvent mixture, significant shifts in peak positions were observed relative to the isolated products described herein.

X-ray Photoelectron Spectroscopy of Polymer Samples.
X-ray photoelectron spectroscopy (XPS) measurements were performed on a Thermo Scientific K-Alpha+ X-ray photoelectron spectrometer using an Al Kα radiation source. Prior to XPS measurements, samples were isolated as a white powder as described above.

Thermal Analysis of Polymer Samples.
Differential scanning calorimetry (DSC) was performed using a Discovery DSC 2500 from TA Instruments. Prior to DSC measurements, samples were dried at 100 °C for 1 hour in an oven. Samples were heated from −30 °C to 200 °C, cooled to −30 °C, and then heated again to 200 °C with a ramp rate of 5 °C/min under nitrogen. The temperature was held constant for two minutes between heating and cool cycles to allow the sample to equilibrate. Glass transition temperatures (T_g) were taken from the second heat cycle. Thermogravimetric Analysis (TGA) was performed on a Discovery TGA 5500 from TA Instruments. TGA measurements included an initial isothermal step (100 °C for 1 hour), followed by cooling to 30 °C and then a temperature ramp to 800 °C at a rate of 10 °C/min under nitrogen. Data was collected after the isothermal hold and then normalized to the starting wt% for this second step.

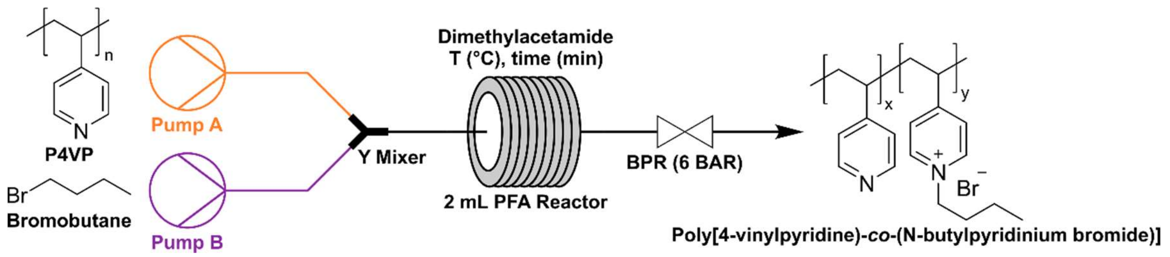

Scheme S1. Continuous Flow Chemistry Setup for Poly[(4-vinylpyridine)-*co*-(N-butylpyridinium bromide)] synthesis.

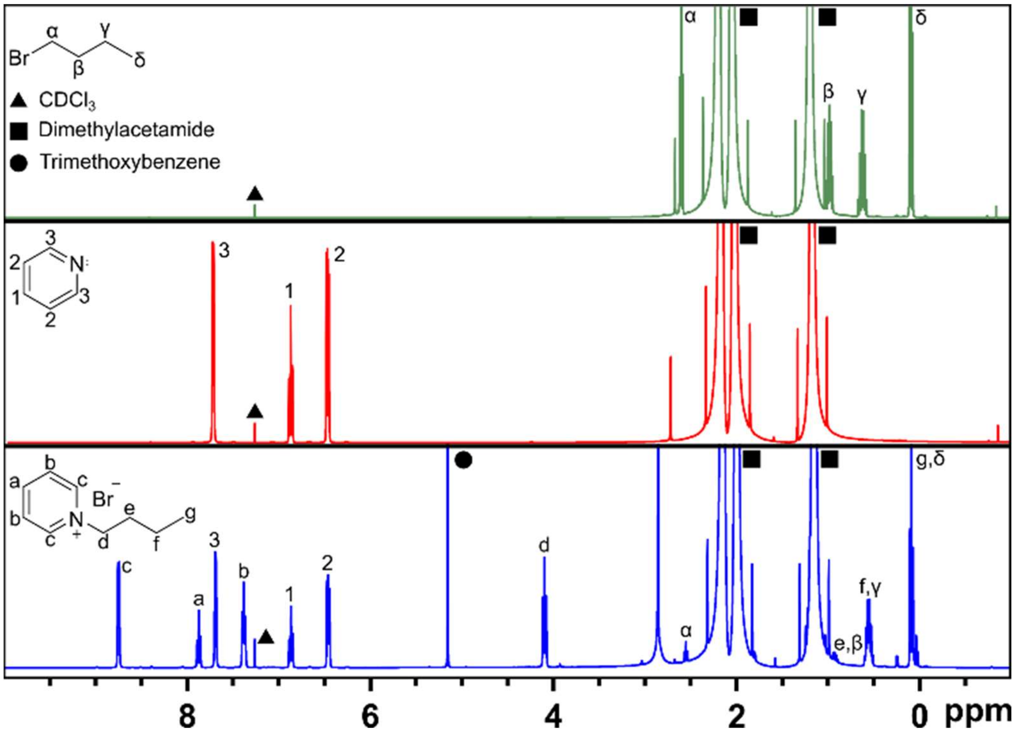

Figure S1. Representative 400 MHz ^{1}H NMR of Butylpyridinium Bromide Product and Reagents. NMR measurements were conducted in a 1:1 v/v mixture of DMAc and $CDCl_3$.

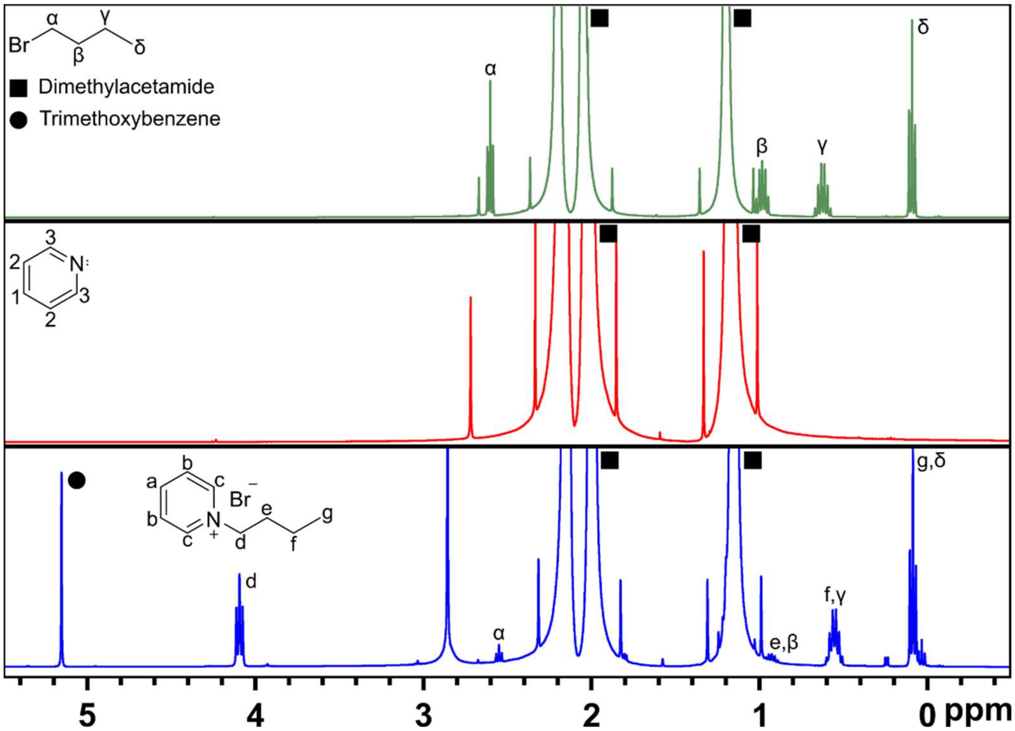

Figure S2. Representative 400 MHz ^{1}H NMR of Butylpyridinium Bromide Product and Reagents in a 1:1 v/v mixture of DMAc and $CDCl_3$. NMR spectral window is expanded to better show peak resolution between 0-5 ppm.

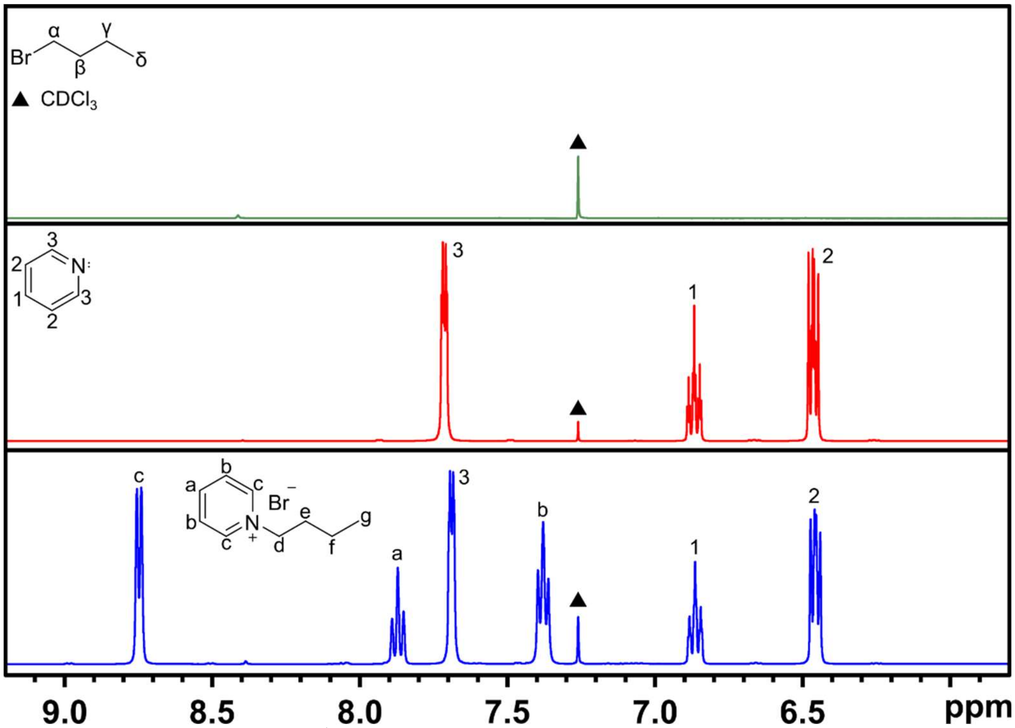

Figure S3. Representative 400 MHz ^{1}H NMR of Butylpyridinium Bromide Product and Reagents in a 1:1 v/v mixture of DMAc and $CDCl_3$. NMR spectral window is expanded to better show peak resolution between 6-9 ppm.

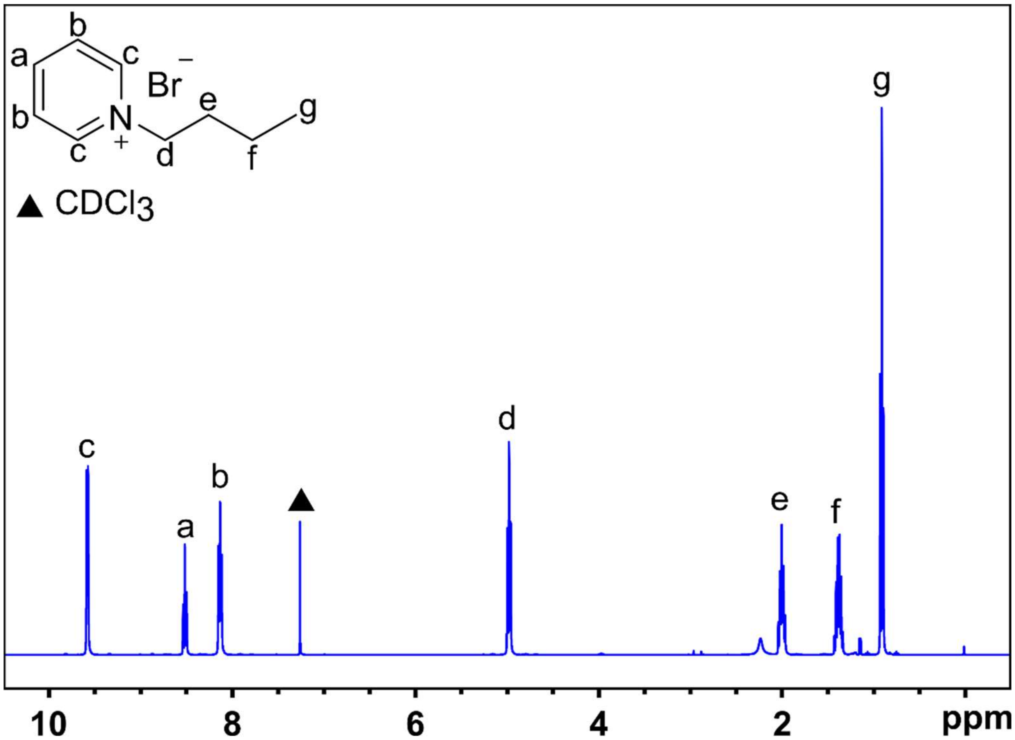

Figure S4. Representative 400 MHz ^{1}H NMR of Isolated Butylpyridinium Bromide in $CDCl_3$.

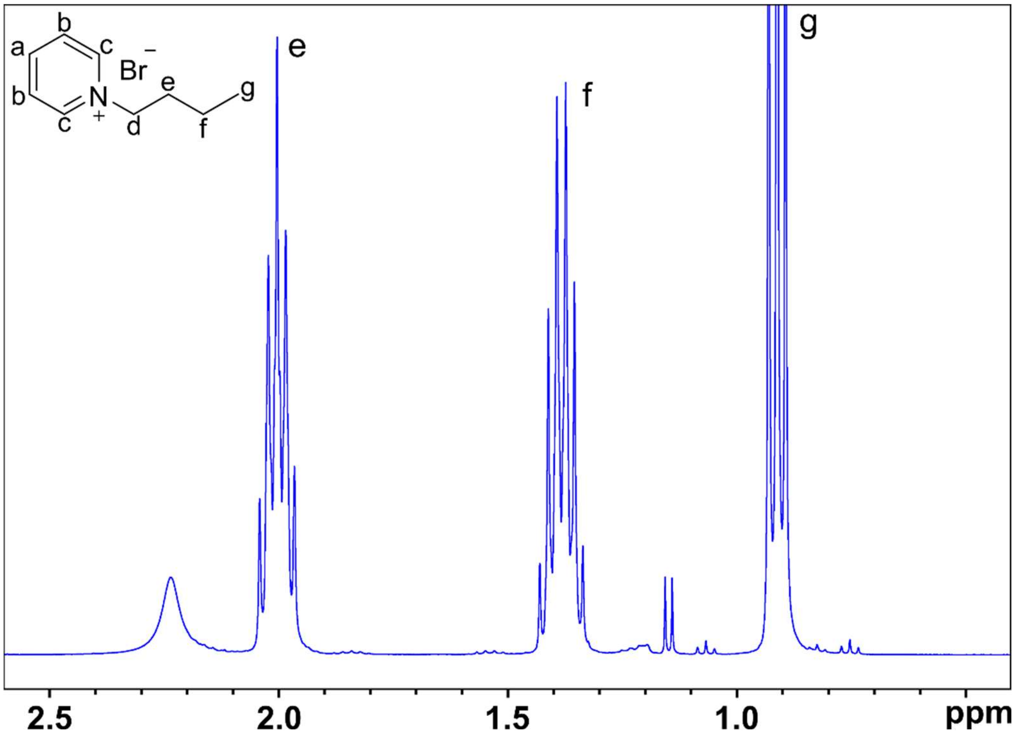

Figure S5. Representative 400 MHz ^{1}H NMR of Isolated Butylpyridinium Bromide in $CDCl_3$. NMR spectral window is expanded to better show peak resolution between 0-2.5 ppm.

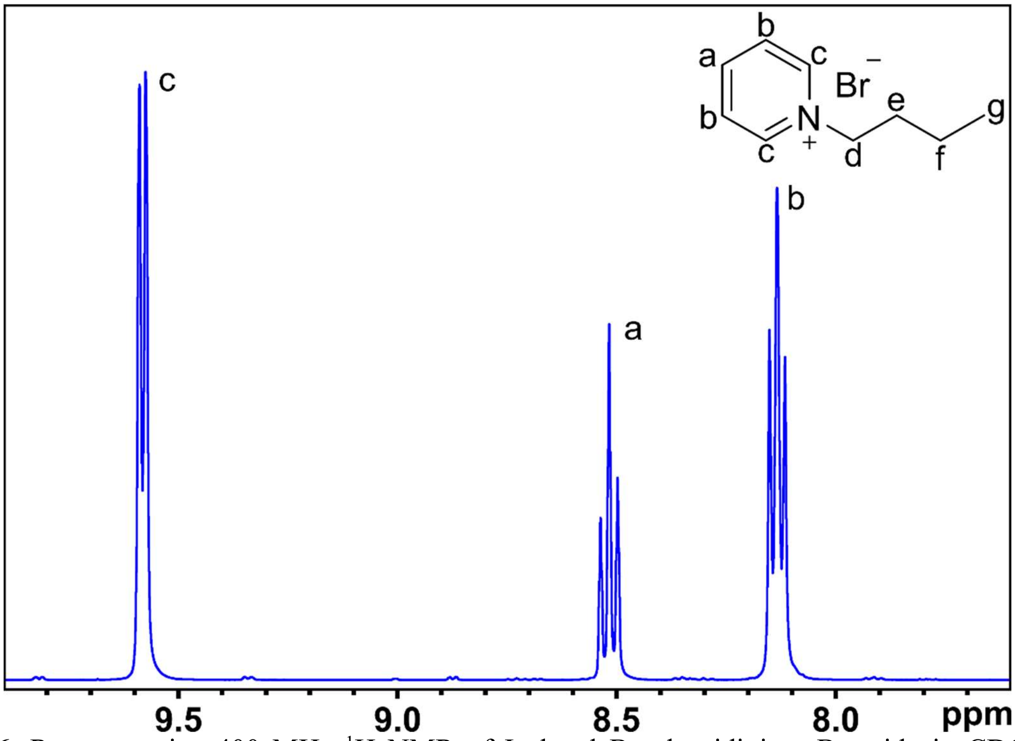

Figure S6. Representative 400 MHz ^{1}H NMR of Isolated Butylpyridinium Bromide in $CDCl_3$. NMR spectral window is expanded to better show peak resolution between 8-9.5 ppm.

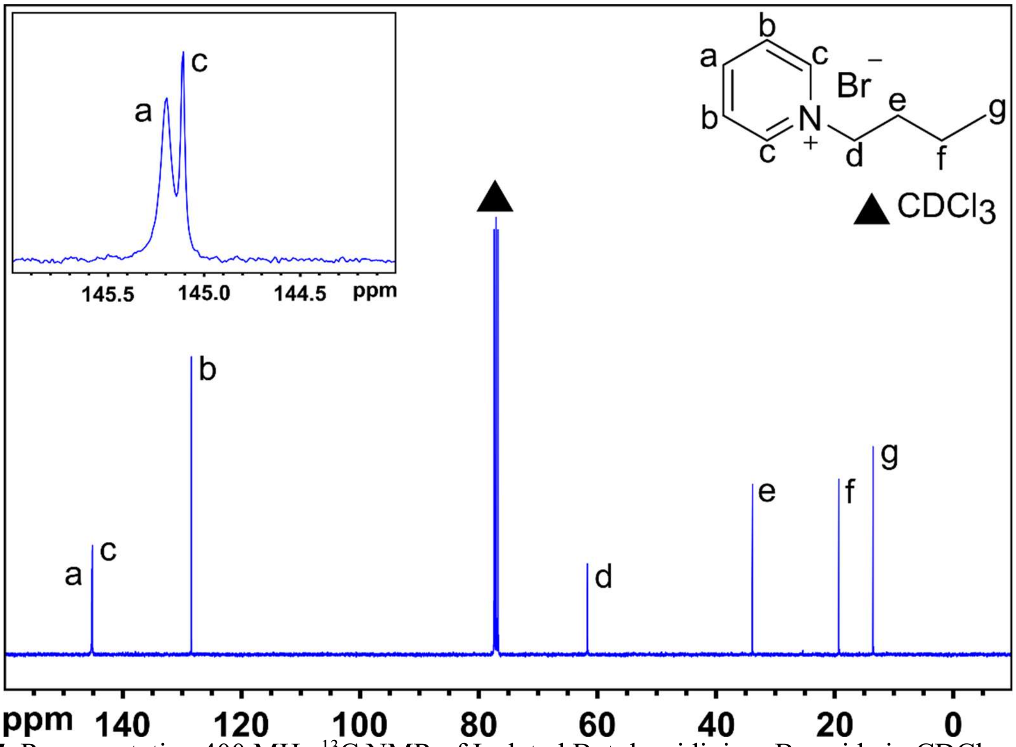

Figure S7. Representative 400 MHz ^{13}C NMR of Isolated Butylpyridinium Bromide in $CDCl_3$.

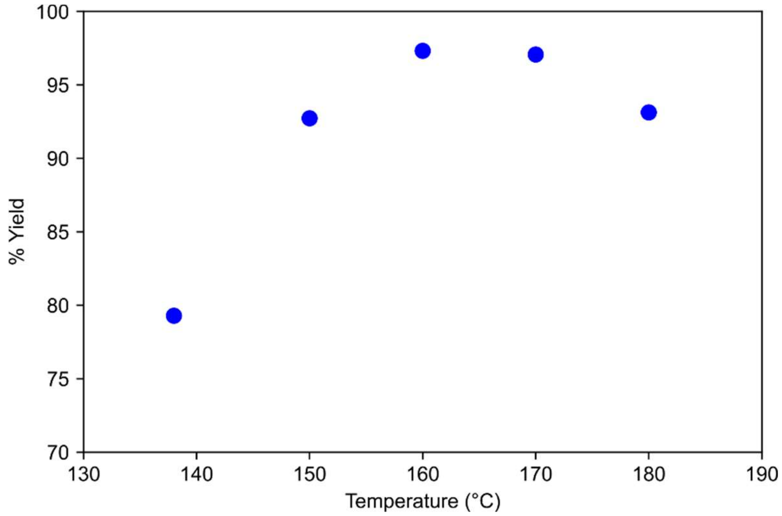

Figure S8. Reaction Yield as a Function of Temperature above 138 °C. The first data point corresponds to 138 °C. Reactions were conducted in a stainless steel (SS) tube reactor capable of temperatures up to 250 °C and pressures up to 42 bar.

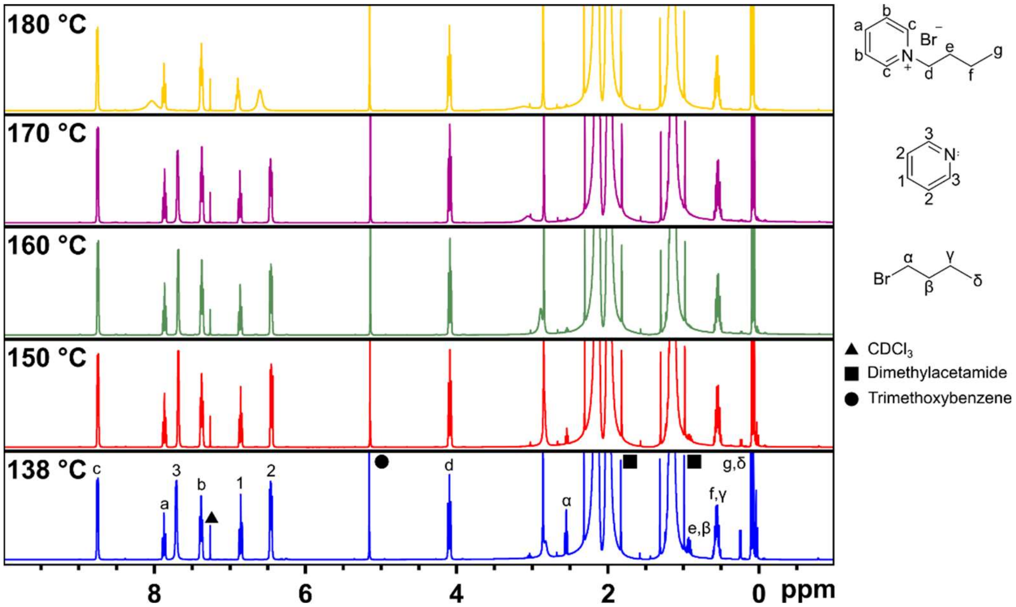

Figure S9. ^{1}H NMR (400 MHz) of Butylpyridinium Bromide as a Function of Temperature. Samples were prepared from crude product solutions in a 1:1 v/v mixture of DMAc and $CDCl_3$.

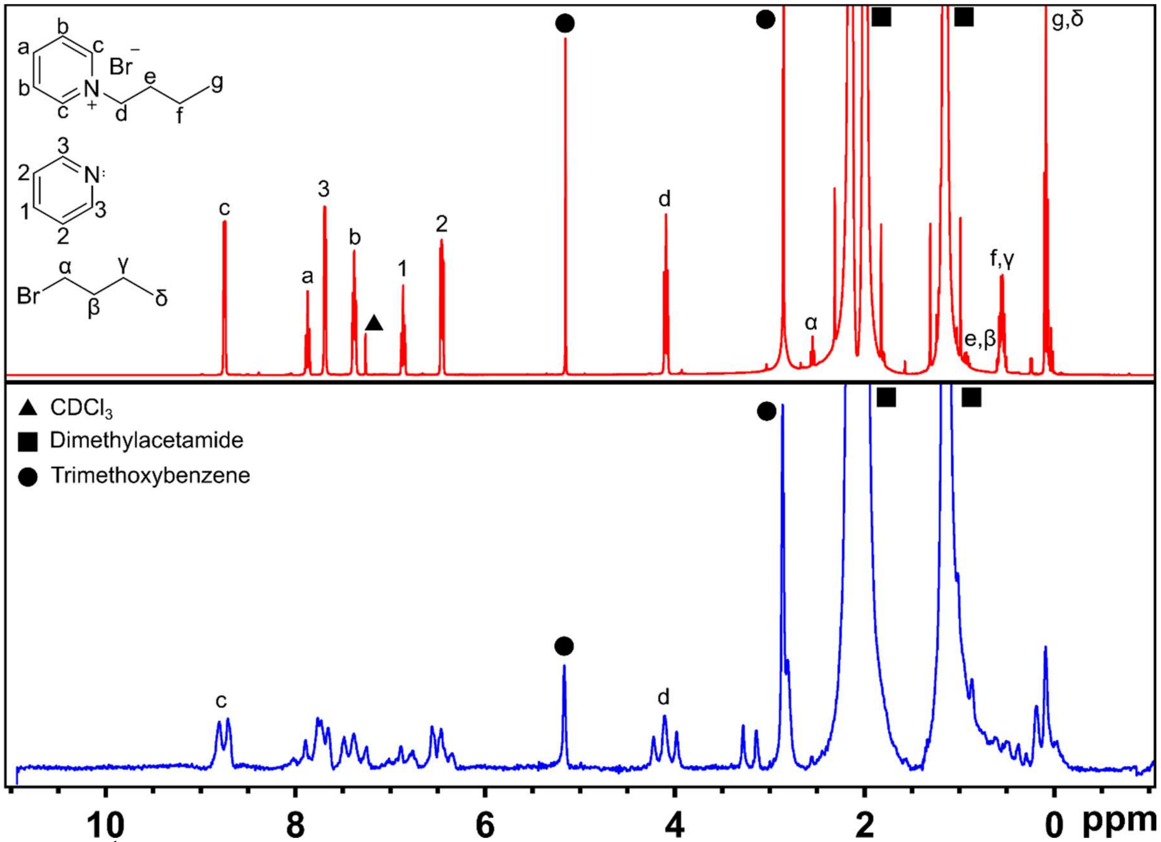

Figure S10. ^{1}H NMR of Representative Butylpyridinium Bromide Product Measured on 400 MHz (top, red) and 60 MHz (bottom, blue) NMR Instruments.

Table S1. Input Parameters and Output Values for the EDBO+ Reaction Campaign. Rounds were conducted as a set of three experiments each, with the exception of an initial seed round. Suggested inputs were based on outputs of the previous rounds. Rounds 11-15 took place after expansion of the upper temperature limit to 168 °C. Yield, Production Rate, and Space-Time Yield are calculated based on 400 MHz manually processed NMR data.

Round	Reaction Number	Temp (°C)	Residence Time (min)	X_{Pyr}	Flow Rate[a] (mL/min)	Yield (%)	Production Rate (g/h)	Space-Time Yield (mmol·mL^{-1}·hr^{-1})
Seed	1	85	23	0.50	0.218	17.78	0.251	0.232
	2	85	23	0.50	0.218	15.23	0.215	0.199
	3	85	23	0.50	0.218	13.92	0.196	0.182
	4	85	23	0.50	0.218	13.21	0.186	0.172
1	1	135	23	0.36	0.218	77.16	0.779	0.721
	2	132	3	0.36	1.667	21.22	1.638	1.516
	3	123	23	0.46	0.217	56.93	0.733	0.678
2	1	138	15	0.56	0.333	67.86	1.295	1.198
	2	138	13	0.63	0.384	70.98	1.309	1.211
	3	135	29	0.63	0.173	87.60	0.725	0.670
3	1	138	41	0.48	0.122	81.59	0.624	0.577
	2	138	5	0.66	1.000	41.12	1.824	1.688
	3	135	1	0.63	5.000	9.252	2.220	2.054
4	1	138	21	0.66	0.238	85.86	0.902	0.835
	2	138	3	0.66	1.667	26.38	1.950	1.805
	3	135	13	0.61	0.385	62.90	1.231	1.139
5	1	138	15	0.48	0.333	66.05	1.373	1.270
	2	138	9	0.48	0.556	51.14	1.771	1.639
	3	138	5	0.51	1.000	32.96	2.105	1.948
6	1	138	29	0.66	0.172	86.31	0.662	0.613
	2	75	3	0.58	1.667	1.61	0.146	0.135
	3	51	1	0.56	5.000	1.73	0.496	0.459
7	1	63	33	0.56	0.152	8.49	0.074	0.068
	2	54	41	0.51	0.122	6.10	0.047	0.044
	3	45	35	0.61	0.143	5.62	0.041	0.038
8	1	138	15	0.43	0.333	73.57	1.366	1.264
	2	138	13	0.48	0.385	62.52	1.498	1.387
	3	138	1	0.33	5.000	10.32	2.208	2.043
9	1	138	33	0.63	0.151	90.24	0.658	0.609
	2	138	23	0.41	0.217	77.41	0.885	0.819
	3	138	13	0.41	0.385	69.79	1.429	1.322
10	1	138	19	0.56	0.263	76.14	1.146	1.061
	2	138	11	0.58	0.455	64.76	1.603	1.483
	3	138	7	0.56	0.714	49.38	2.012	1.862
11	1	168	17	0.48	0.293	78.98	1.450	1.342
	2	168	11	0.60	0.453	87.15	2.053	1.899
	3	165	5	0.33	1.000	69.11	2.958	2.737
12	1	168	1	0.39	5.000	22.14	5.601	5.183
	2	168	1	0.36	4.990	22.18	5.160	4.775
	3	165	3	0.57	1.666	44.54	4.137	3.829
13	1	165	43	0.51	0.115	82.89	0.609	0.563
	2	162	41	0.51	0.121	80.78	0.623	0.577
	3	159	41	0.48	0.121	82.84	0.628	0.581
14	1	168	9	0.57	0.554	81.45	2.521	2.333
	2	168	5	0.60	1.000	67.80	3.517	3.254
	3	168	3	0.63	1.666	52.44	4.191	3.878
15	1	168	27	0.54	0.185	84.83	0.936	0.866
	2	156	29	0.66	0.171	94.48	0.716	0.663
	3	156	29	0.33	0.171	89.93	0.659	0.609

[a]Flow Rate refers to the combined total flow rate of pyridine and bromobutane.

Table S2. Hyperparameter values in the surrogate models after Round 10 (upper temperature bound of 138 °C). Smaller values indicate a greater contribution to the output objective

Model Number	Objective	Samples	Noise	θ_{Temp}	θ_{Time}	$\theta_{mole\ fraction\ pyridine}$
0	Production rate ($g \cdot h^{-1}$)	34 (thru Rnd 10)	0.021790	0.957210	1.478469	10.390768
1	Yield (%)	34 (thru Rnd 10)	0.012201	1.524354	1.032122	11.879192

Table S3. Hyperparameter values in the surrogate models after Round 15 (upper temperature bound of 168 °C). Smaller values indicate a greater contribution to the output objective.

Model Number	Objective	Samples	Noise	θ_{Temp}	θ_{Time}	$\theta_{mole\ fraction\ pyridine}$
0	STY ($mmol \cdot mL^{-1} \cdot h^{-1}$)	49 (thru Rnd 15)	0.010221	0.793552	0.470286	15.050981
1	Yield (%)	49 (thru Rnd 15)	0.013063	0.736270	0.526247	13.977031

Table S4. Comparison of Output Values Calculated From Low- and High-Field NMR Data. Outputs were calculated via manual processing in Bruker TopSpin Software (blue, black) are compared to those calculated using semi-automated nmrglue processing on the same samples (green, purple).

Round	Reaction Number	Temp (°C)	τ_{Res} (min)	X_{Pyr}	Yield (%)				Production Rate (g/h)				Space-Time Yield (mmol·mL^{-1}·h^{-1})			
					60 MHz	60 MHz	400 MHz	400 MHz	60 MHz	60 MHz	400 MHz	400 MHz	60 MHz	60 MHz	400 MHz	400 MHz
					Manual	Auto	Manual	Auto	Manual	Auto	Manual	Auto	Manual	Auto	Manual	Auto
1	1	135	23	0.36	63.45	78.80	77.16	75.77	0.640	0.795	0.779	0.765	0.593	0.736	0.721	0.708
	2	132	3	0.36	15.41	18.45	21.22	8.67	1.189	1.424	1.638	0.669	1.101	1.318	1.516	0.619
	3	123	23	0.46	71.72	57.13	56.93	55.43	0.923	0.735	0.733	0.713	0.854	0.680	0.678	0.660
2	1	138	15	0.56	76.42	70.28	67.86	67.66	1.458	1.341	1.295	1.291	1.349	1.241	1.198	1.195
	2	138	13	0.63	60.87	69.36	70.98	70.93	1.123	1.279	1.309	1.308	1.039	1.184	1.211	1.211
	3	135	29	0.63	91.99	86.04	87.60	88.04	0.761	0.712	0.725	0.728	0.704	0.659	0.670	0.674
3	1	138	41	0.48	93.28	86.49	81.59	78.62	0.713	0.661	0.624	0.601	0.660	0.612	0.577	0.556
	2	138	5	0.66	62.05	37.37	41.12	39.79	2.753	1.658	1.824	1.765	2.547	1.534	1.688	1.633
	3	135	1	0.63	6.34	4.60	9.252	0.02	1.521	1.103	2.220	0.006	1.408	1.021	2.054	0.005
4	1	138	21	0.66	80.38	85.08	85.86	82.95	0.845	0.894	0.902	0.872	0.782	0.828	0.835	0.807
	2	138	3	0.66	29.26	17.15	26.38	10.21	2.163	1.267	1.950	0.755	2.001	1.173	1.805	0.699
	3	135	13	0.61	75.21	66.04	62.90	60.96	1.471	1.292	1.231	1.193	1.362	1.196	1.139	1.104
5	1	138	15	0.48	71.67	67.23	66.05	65.63	1.490	1.397	1.373	1.364	1.379	1.293	1.270	1.262
	2	138	9	0.48	55.14	48.98	51.14	50.77	1.909	1.696	1.771	1.758	1.767	1.570	1.639	1.627
	3	138	5	0.51	39.66	30.63	32.96	32.25	2.532	1.956	2.105	2.059	2.344	1.810	1.948	1.906
6	1	138	29	0.66	94.65	86.37	86.31	85.59	0.726	0.662	0.662	0.656	0.672	0.613	0.613	0.607
	2	75	3	0.58	0.38	-2.26	1.61	-0.15	0.034	-0.205	0.146	-0.013	0.032	-0.190	0.135	-0.012
	3	51	1	0.56	0.83	-2.22	1.73	1.71	0.239	-0.637	0.496	0.490	0.221	-0.590	0.459	0.454
7	1	63	33	0.56	12.25	2.70	8.49	0.02	0.106	0.023	0.074	0.000	0.098	0.022	0.068	0.000
	2	54	41	0.51	4.92	1.91	6.10	-0.03	0.038	0.015	0.047	0.000	0.035	0.014	0.044	0.000
	3	45	35	0.61	6.22	0.47	5.62	-0.15	0.045	0.003	0.041	-0.001	0.042	0.003	0.038	-0.001
8	1	138	15	0.43	70.84	72.43	73.57	73.47	1.315	1.344	1.366	1.364	1.217	1.244	1.264	1.262
	2	138	13	0.48	86.38	65.08	62.52	61.94	2.070	1.560	1.498	1.485	1.916	1.443	1.387	1.374
	3	138	1	0.33	8.54	6.97	10.32	-0.11	1.827	1.492	2.208	-0.022	1.691	1.381	2.043	-0.021
9	1	138	33	0.63	92.46	113.37	90.24	88.06	0.674	0.826	0.658	0.642	0.623	0.764	0.609	0.594
	2	138	23	0.41	87.32	92.16	77.41	76.48	0.998	1.054	0.885	0.874	0.924	0.975	0.819	0.809
	3	138	13	0.41	92.08	86.40	69.79	68.41	1.885	1.769	1.428	1.400	1.744	1.637	1.322	1.296
10	1	138	19	0.56	98.37	80.06	76.14	75.49	1.481	1.205	1.146	1.136	1.370	1.115	1.061	1.052
	2	138	11	0.58	80.75	60.34	64.76	64.34	1.998	1.493	1.603	1.592	1.849	1.382	1.483	1.473
	3	138	7	0.56	58.02	47.92	49.38	49.07	2.364	1.952	2.012	1.999	2.187	1.806	1.862	1.850
11	1	168	17	0.48	68.51	91.07	78.98	78.27	1.257	1.672	1.450	1.437	1.164	1.547	1.342	1.330
	2	168	11	0.60	93.65	84.93	87.15	82.39	2.206	2.000	2.053	1.940	2.041	1.851	1.899	1.796
	3	165	5	0.33	70.39	76.66	69.11	69.14	3.013	3.281	2.958	2.959	2.788	3.036	2.737	2.738
12	1	168	1	0.39	23.16	20.59	22.14	11.13	5.859	5.208	5.601	2.815	5.422	4.820	5.183	2.605
	2	168	1	0.36	26.69	22.72	22.18	10.70	6.210	5.285	5.160	2.488	5.747	4.891	4.775	2.303
	3	165	3	0.57	41.91	50.58	44.54	44.42	3.893	4.698	4.137	4.126	3.602	4.348	3.829	3.818
13	1	165	43	0.51	78.19	82.85	82.89	82.78	0.574	0.609	0.609	0.608	0.532	0.563	0.563	0.563
	2	162	41	0.51	80.04	87.54	80.78	80.29	0.617	0.675	0.623	0.619	0.571	0.625	0.577	0.573
	3	159	41	0.48	64.34	81.00	82.84	81.70	0.488	0.614	0.628	0.619	0.451	0.568	0.581	0.573
14	1	168	9	0.57	64.92	84.99	81.45	81.72	2.009	2.630	2.521	2.529	1.860	2.434	2.333	2.341
	2	168	5	0.60	76.71	67.57	67.80	67.66	3.979	3.505	3.517	3.510	3.682	3.243	3.254	3.248
	3	168	3	0.63	59.13	57.40	52.44	51.91	4.725	4.587	4.191	4.149	4.373	4.245	3.878	3.839
15	1	168	27	0.54	81.60	82.50	84.83	83.20	0.900	0.910	0.936	0.918	0.833	0.842	0.866	0.849
	2	156	29	0.66	91.36	95.97	94.48	102.28	0.693	0.728	0.716	0.776	0.641	0.674	0.663	0.718
	3	156	29	0.33	75.68	99.54	89.93	90.12	0.554	0.729	0.659	0.660	0.513	0.675	0.609	0.611

Table S5. Error in NMR Yield and Space-Time Yield (STY) Determined from Low- and High- Field NMR Data Analysis.

	60 MHz Manual	60 MHz Auto	400 MHz Manual[a]	400 MHz Auto
**Yield Mean Absolute Error**	8.0	4.4	0.0	2.9
**STY Mean Absolute Error**	0.2	0.2	0.0	0.3

[a]Manually processed data from the 400 MHz NMR represent ground truth.

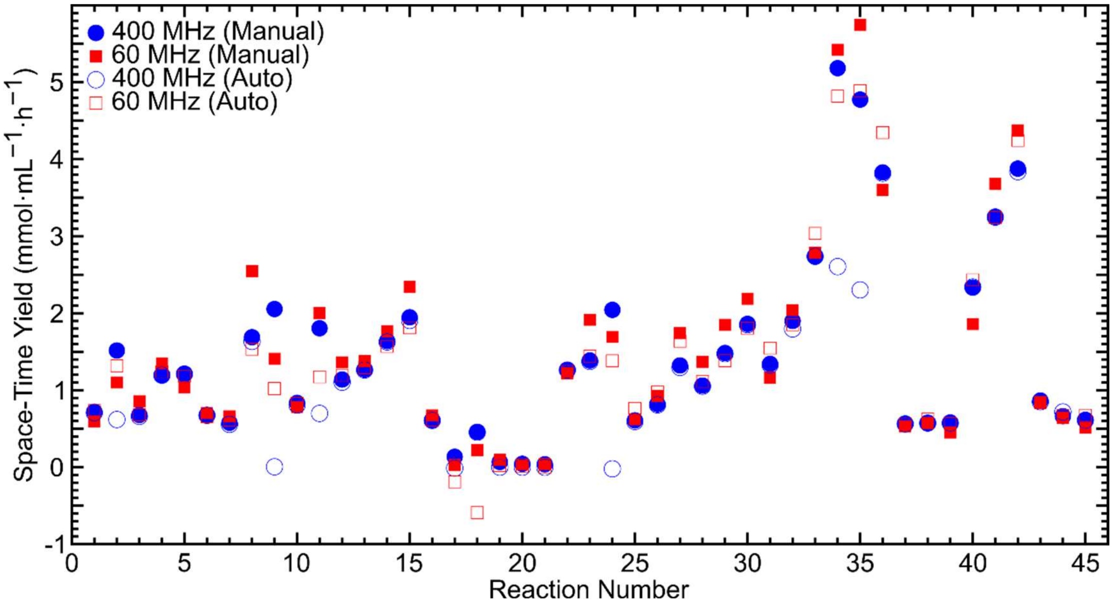

Figure S11. Space-Time Yields Calculated from Manual and Automated Processing Methods.

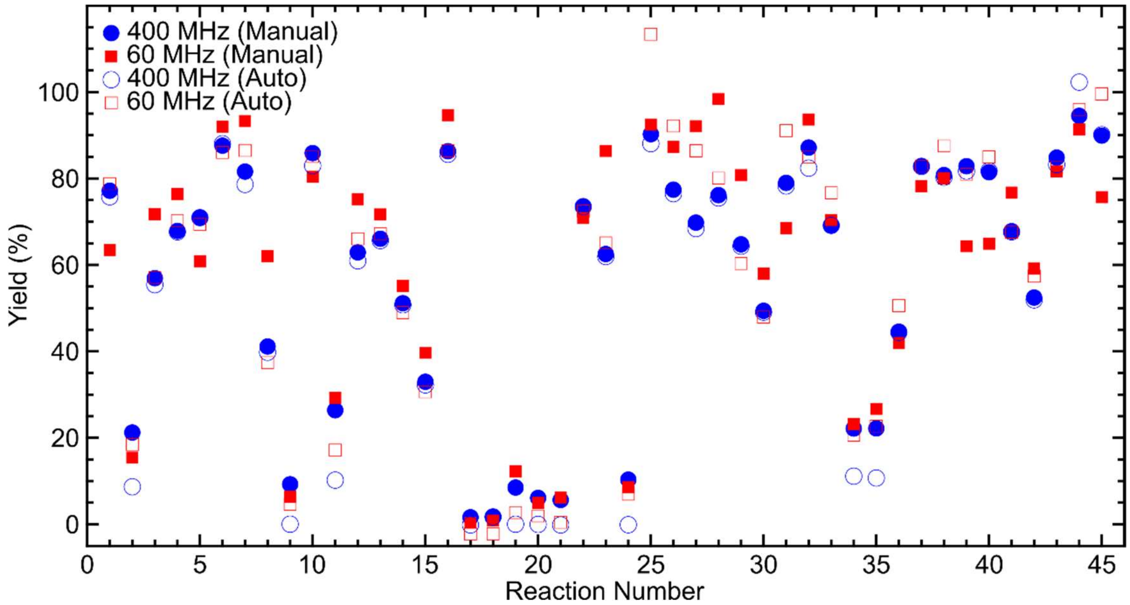

Figure S12. NMR Yields Calculated from Manual and Automated Processing Methods.

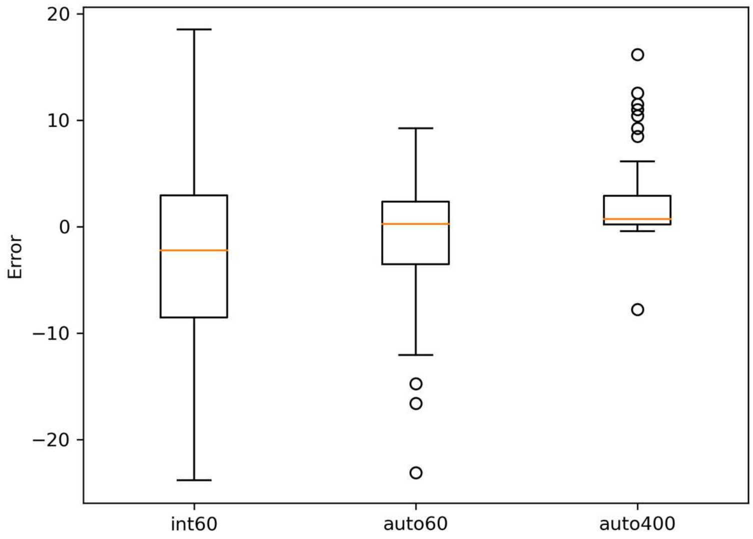

Figure S13. A boxplot of NMR data comparing manual integration from the 60 MHz instrument and the semi-automated integrations from 60 and 400 MHz NMR data. The median of the data and outliers are represented by orange lines and black circles, respectively. The boxplot is a graphical representation of the spread and skewness of the NMR integrations through their corresponding quartiles.

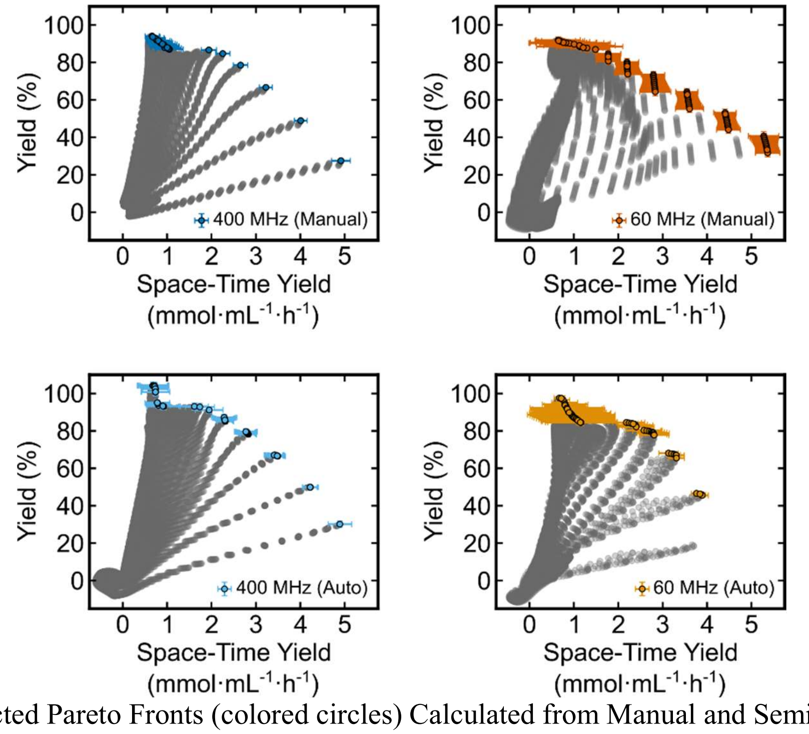

Figure S14. Predicted Pareto Fronts (colored circles) Calculated from Manual and Semi-Automated Processing of Low- and High-Field NMR Data. Error bars represent a 95% confidence interval. Grey circles represent dominated predictions including previously run data points.

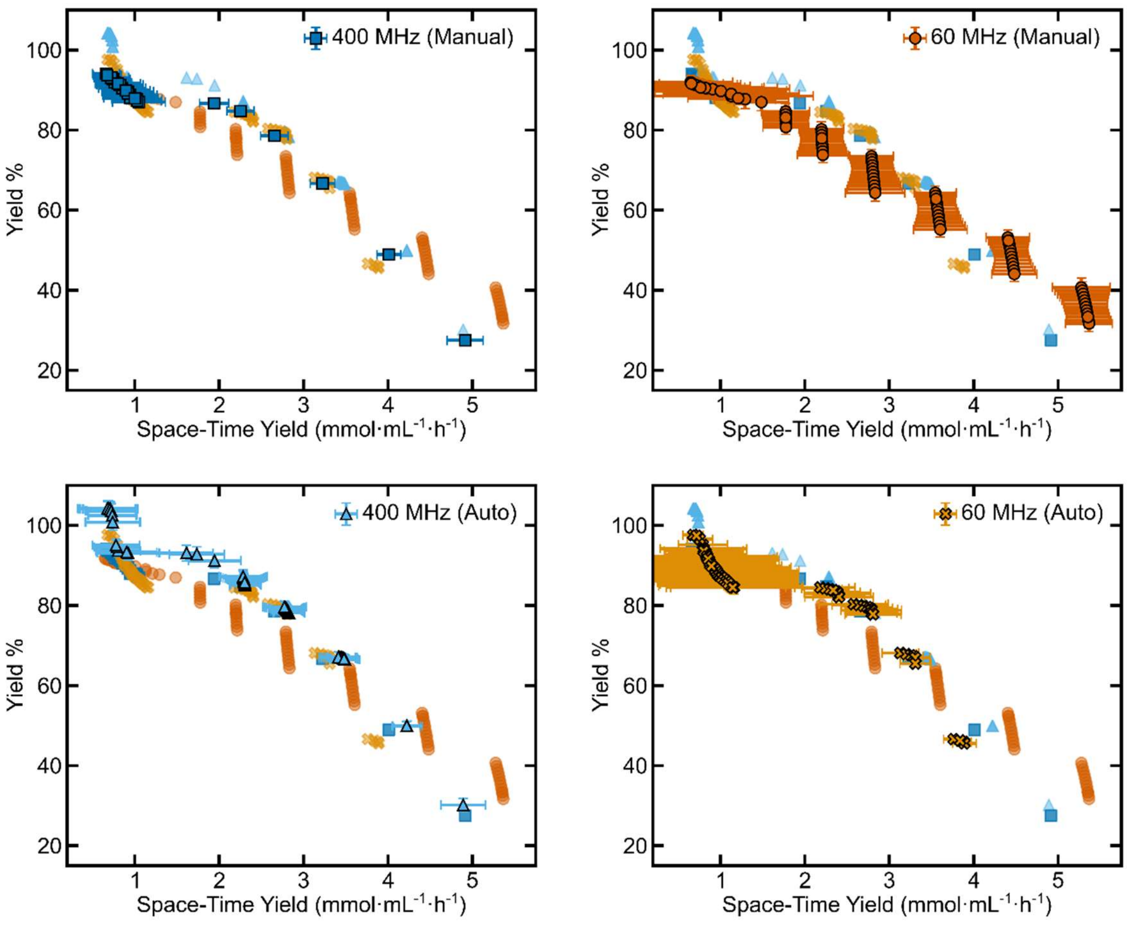

Figure S15. Predicted Pareto Fronts From All Analytical Methods. Error bars indicate a 95% confidence interval.

Table S6. Predicted Maximum Hypervolume of the Pareto Front After Round 15 for Each NMR Analysis Method. Hypervolume predictions contain no experimental data.

NMR Analysis Method	Predicted Hypervolume (% Yield·(mmol·mL^{-1}·h^{-1}))
400 MHz Manual	334
60 MHz Manual	370
400 MHz Auto	308
60 MHz Auto	363

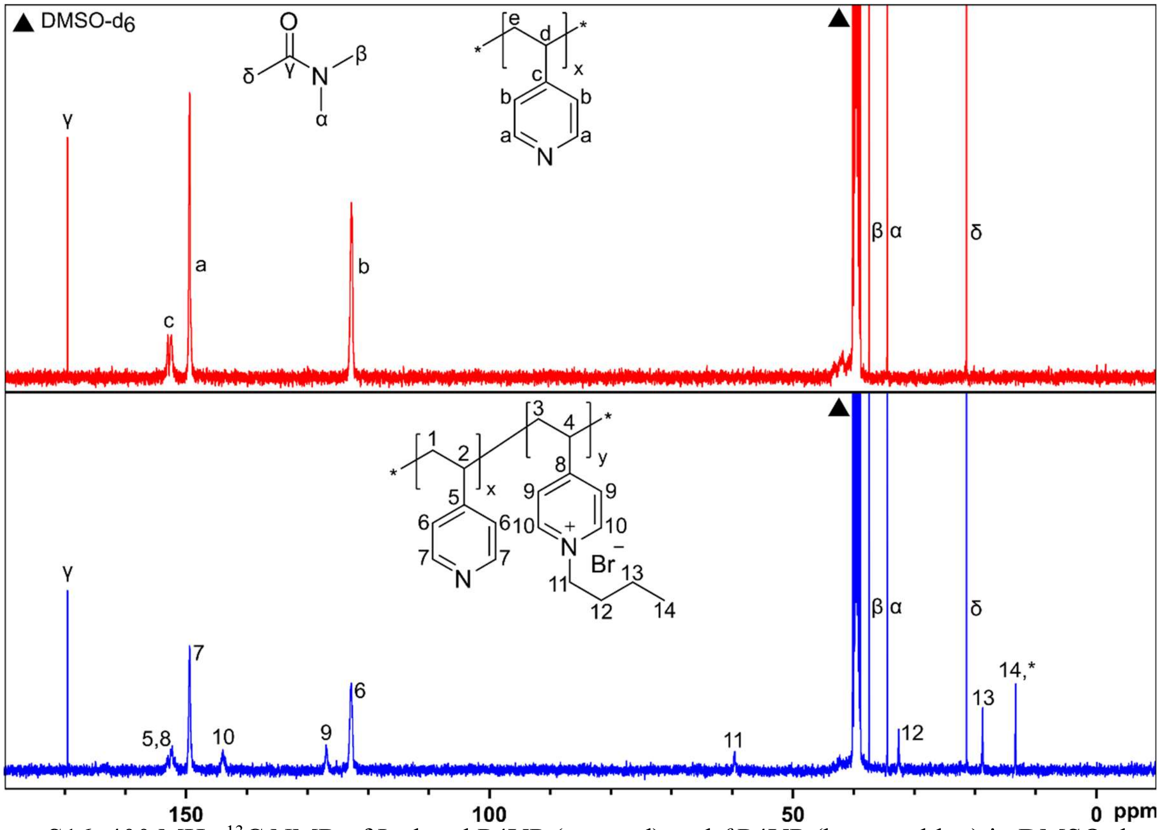

Figure S16. 400 MHz ^{13}C NMR of Isolated P4VP (top, red) and *f*-P4VP (bottom, blue) in DMSO-d_6.

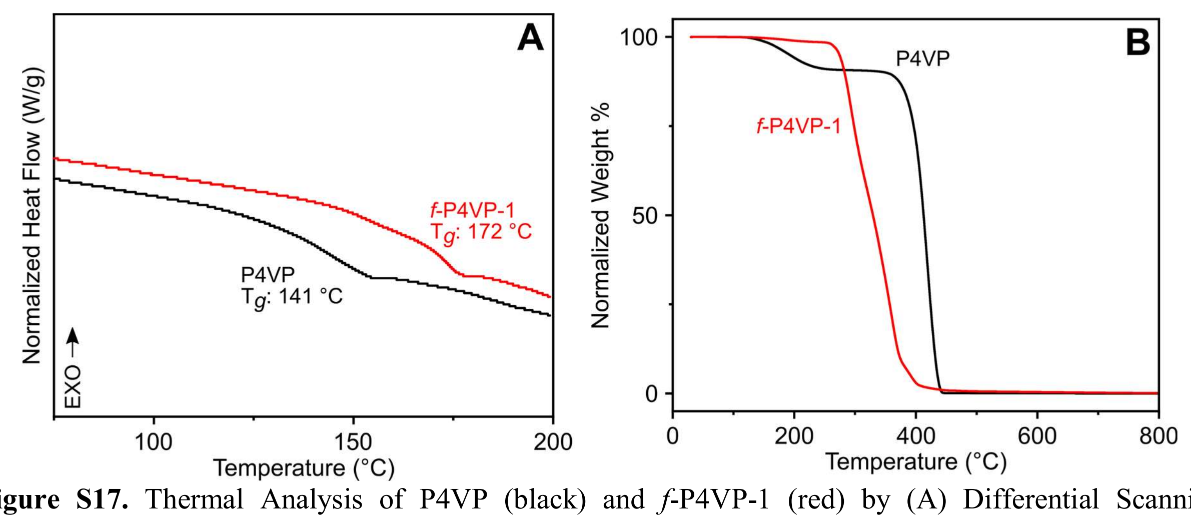

Figure S17. Thermal Analysis of P4VP (black) and *f*-P4VP-1 (red) by (A) Differential Scanning Calorimetry (DSC) and (B) Thermogravimetric Analysis (TGA). DSC Thermograms were obtained from a single measurement.

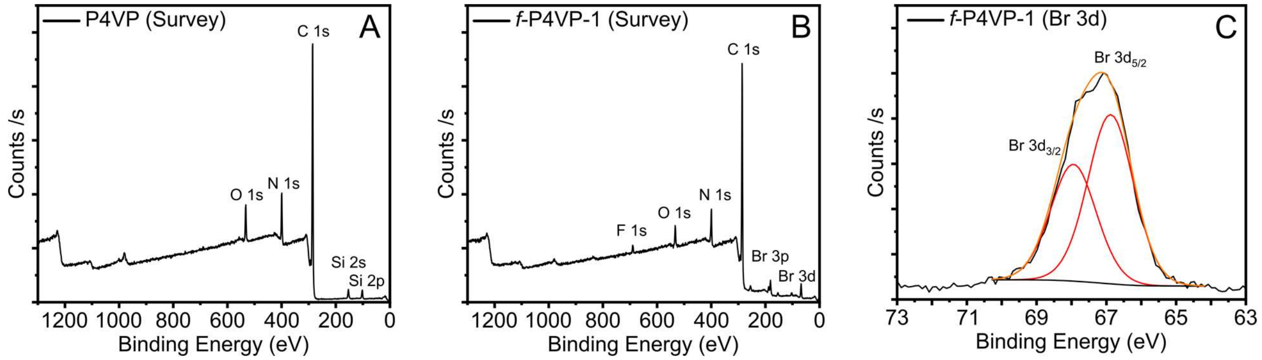

Figure S18. X-ray Photoelectron Spectroscopy (XPS) of Polymer Samples. (A) Survey spectrum of isolated P4VP, (B) Survey spectrum of *f*-P4VP-1, and (C) Br 3d spectrum of *f*-P4VP-1. All samples were isolated from solutions in DMAc as described above.

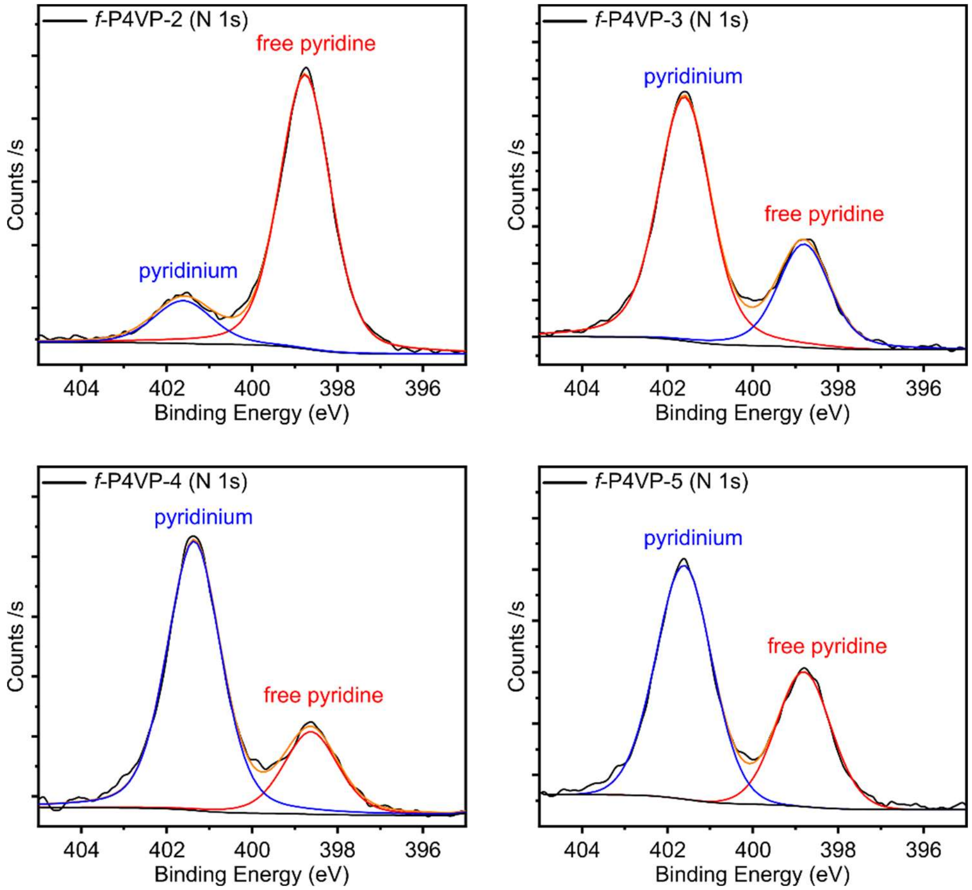

Figure S19. X-ray Photoelectron Spectroscopy (XPS) of *f*-P4VP Samples Prepared in Batch. Reactions were conducted in batch using conditions suggested by EDBO+ during the pyridine reaction campaign, with the exception of *f*-P4VP-5 for which the reaction parameters were chosen from previously untested inputs.

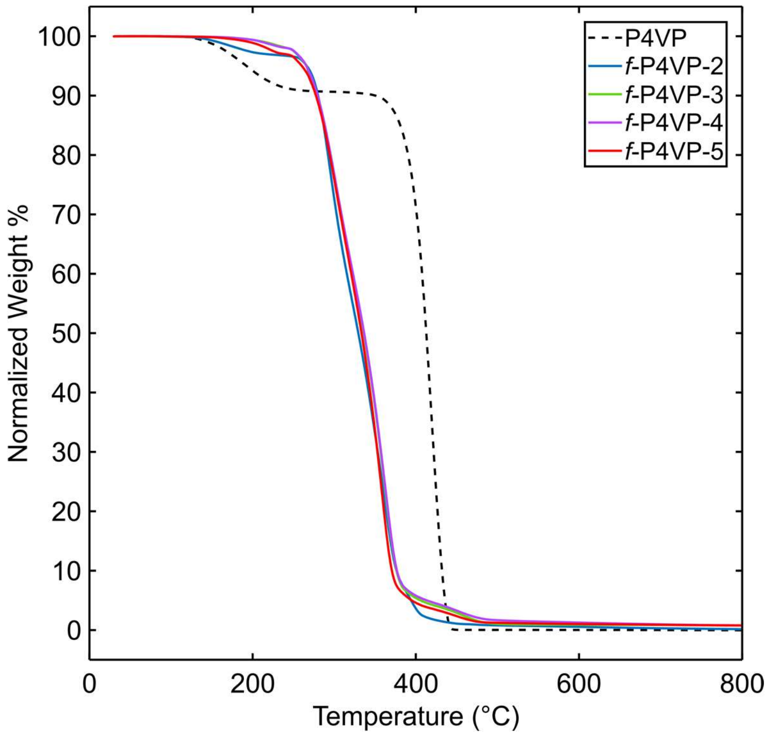

Figure S20. Thermogravimetric Analysis (TGA) of *f*-P4VP Samples Prepared in Batch.

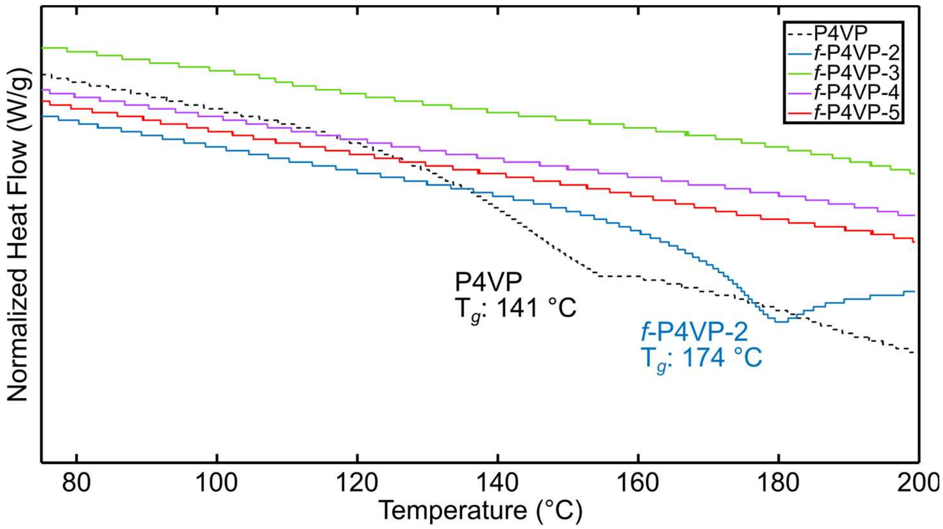

Figure S21. Differential Scanning Calorimetry (DSC) of *f*-P4VP Samples Prepared in Batch.

Table S7. Comparison of *f*-P4VP Quaternization Yields Calculated from ^{1}H NMR and XPS Data from Batch Reactions.

Sample	Small Molecule Analogue[a] Yield (%)	^{1}H NMR (%)	XPS[b] (%)
***f*-P4VP-1**[c]	9.25	16.30	11.94
***f*-P4VP-2**	17.78	17.48	15.20
***f*-P4VP-3**	77.41	77.39	70.60
***f*-P4VP-4**	90.24	78.16	76.50
***f*-P4VP-5**[d]	59.88	68.18	64.10

[a]Reaction yield of the small molecule analogue synthesized in flow from EDBO+ suggested inputs.
[b]Degree of quaternization corresponds to the atomic % of the pyridinium nitrogen in the XPS N1s spectrum.
[c]The functionalization of *f*-P4VP-1 was conducted in flow and discussed in the main text.
[d]Reaction conditions were selected that were previously untested during the small molecule reaction campaign. The small molecule analogue yield was predicted by EDBO+ given reaction conditions of 123 °C, 23 min τ_{res}, and 0.42 χ_{Pyr}. This demonstrated that the predicted yield of a reaction may be close to experimental values.

References

1 J. A. G. Torres, S. H. Lau, P. Anchuri, J. M. Stevens, J. E. Tabora, J. Li, A. Borovika, R. P. Adams and A. G. Doyle, *J. Am. Chem. Soc.*, , DOI:10.1021/jacs.2c08592.

2 B. J. Shields, J. Stevens, J. Li, M. Parasram, F. Damani, J. I. M. Alvarado, J. M. Janey, R. P. Adams and A. G. Doyle, *Nature*, 2021, **590**, 89–96.

3 J. J. Helmus and C. P. Jaroniec, *J. Biomol. NMR*, 2013, **55**, 355–367.
